# Single Molecular Layer of Chitin Sub‐Nanometric Nanoribbons: One‐Pot Self‐Exfoliation and Crystalline Assembly into Robust, Sustainable, and Moldable Structural Materials

**DOI:** 10.1002/advs.202201287

**Published:** 2022-03-31

**Authors:** Yugao Ding, Xizhi Chen, Youshuang Zhou, Xiaoming Ren, Weihua Zhang, Mingjie Li, Qunchao Zhang, Tao Jiang, Beibei Ding, Dean Shi, Jun You

**Affiliations:** ^1^ Key Laboratory for the Green Preparation and Application of Functional Materials Hubei Key Laboratory of Polymer Materials School of Materials Science and Engineering Hubei University Youyi Road 368 Wuhan 430062 China; ^2^ Key Laboratory for Deep Processing of Major Grain and Oil Wuhan Polytechnic University Ministry of Education Wuhan 430023 China; ^3^ CAS Key Lab of Bio‐Based Materials Qingdao Institute of Bioenergy and Bioprocess Technology Chinese Academy of Sciences Songling Road 189 Qingdao 266101 P. R. China

**Keywords:** chitin nanoribbons, pseudosolvent, self‐exfoliation, single molecular layer, sub‐nanometric materials

## Abstract

Sub‐nanometric materials (SNMs) represent a series of unprecedented size‐/morphology‐related properties applicable in theoretical research and diverse cutting‐edge applications. However, in‐depth investigation and wide utilization of organic SNMs are frequently hindered, owing to the complex synthesis procedures, insufficient colloidal stability, poor processability, and high cost. In this work, a low‐cost, energy‐efficient, convenient, effective, and scalable method is demonstrated for directly exfoliating chitin SNMs from their natural sources through a one‐pot “tandem molecular intercalation” process. The resultant solution‐like sample, which exhibits ribbon‐like feature and contains more than 85% of the single molecular layer (thickness <0.6 nm), is capable of being solution‐processed to different types of materials. Thanks to the sub‐nanometric size and rich surface functional groups, chitin SNMs reveal versatile intriguing properties that rarely observe in their nano‐counterparts (nanofibrils), e.g., crystallization‐like assembly in the colloidal state and alcoplasticity/self‐adhesiveness in the bulk aggregate state. The finding in this work not only opens a new avenue for the high value‐added utilization of chitin, but also provides a new platform for both the theoretical study and practical applications of organic SNMs.

## Graphical abstract

A one‐pot “tandem molecular intercalation” process is developed to efficiently produce chitin sub‐nanometric nanoribbons with unprecedented >85% of single molecular layer. Thanks to the sub‐nanometric size and rich surface functional groups, these nanoribbons reveal versatile intriguing properties that rarely observed in their nano‐counterparts (nanofibrils), and thus provide new platform for both theoretical study and practical applications of organic sub‐nanometric materials.

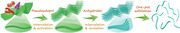

## 1 Introduction

Sub‐nanometric materials (SNMs) refer to nanomaterials with at least one of their dimensions that reaches atomic or single molecular level, which is capable of bridging the gap between traditional nanomaterials and molecules.^[^ ^1^ ^]^ Compared with their larger‐sized nano‐counterparts, SNMs represent a series of unprecedented size‐/morphology‐related physical/chemical properties, which not only have far‐reaching implications for theoretical research, but also show great promise in diverse cutting‐edge applications such as catalysis, energy conversion, optics, and ion separation.^[^ ^2^ ^]^ The most archetypal and pioneering sub‐nanometric material must be graphene; it has enjoyed a great deal of studies on its monatomic structure and intriguing properties, and so do other inorganic/carbon SNMs such as carbon nitride, black phosphorus, transition metal dichalcogenides,and MXenes.^[^ ^3^ ^]^ Their successful and widespread applications have incentivized and laid a fertile ground for the rapid development of organic SNMs (e.g., 2D polymers). Unlike inorganic/carbon SNMs, monolayered organic SNMs have customizable structures and functionality by selecting diverse building blocks or postmodifying functional groups, and thereby have become focal point in polymer research.^[^ ^4^ ^]^

Bottom‐up strategies, such as on‐surface and topochemical polymerization, have been first applied to produce organic SNMs since 1989.^[^ ^5^ ^]^ Through the confined polymerization/crosslinking of preassembled monomers between two phase interfaces (e.g., air/water interface), large‐area organic nanofilms with monomolecular thickness can be directly synthesized. However, after decades of development, these methods are still limited for widespread applications, on account of their tedious synthesis procedures (specifically designed monomers), rigorous reaction conditions (ultrahigh vacuum), high cost (expensive substrates), and low productivity.^[^ ^4a^ ^]^ In order to solve these problems, a top‐down exfoliation method is inspired from inorganic 2D materials and utilized to produce organic SNMs (i.e., 2D polymers). This approach involves the topochemical synthesis of layered polymer crystals, in combination with solvent‐diffusion and external mechanical treatments (e.g., sonication and agitation).^[^ ^6^ ^]^ The selective cleavage of interlayer secondary forces is the key factor for the exfoliation of atomically thin and even single‐layered organic SNMs. Therefore, these polymer crystals, which should have robust intralayer covalent/coordinate linkage while weak interlayer interactions (e.g., van der Waals and hydrogen bonding), must be meticulously designed. Over the past decade, a series of new synthetic techniques have been developed for the production of lamellar organic crystals, such as single‐crystal to single‐crystal approach^[^ ^7^ ^]^ and 2D covalent organic frameworks (COF) synthesis.^[^ ^8^ ^]^ Despite these contributions, stable and concentrated dispersion with a high proportion of single‐layered organic SNMs, which is capable of being solution‐processed to different types of materials (e.g., films, aerogels, and fibers), keeps far from being in‐depth studied and achieved for wide applications.^[^ ^9^ ^]^

Natural biopolymers, such as cellulose, chitin, and silk, mainly exist in the form of semicrystalline nanofibrils with alternating amorphous chains and nanocrystals.^[^ ^10^ ^]^ Many crystallographic results have revealed that these fibrillar crystals are naturally gifted with lamellar structures that consist of monolayered molecular sheets (Figure S1, Supporting Information).^[^ ^11^ ^]^ Analogous to graphene, they have strong intralayer interactions (intrachain covalent linking and interchain multiple hydrogen bonding) with weak interlayer binding force, which make them a suitable candidate for the exfoliation of organic SNMs. Nonetheless, current researches mainly focus on the exfoliation and application of nanometric intact nanofibrils;^[^ ^12^ ^]^ there are only a few reports about the in‐depth delamination of nanofibrils to monolayered molecular sheets, albeit with a high energy consumption and relatively low yield.^[^ ^13^ ^]^ In our previous work, we reported an original finding that dimethylsulfoxide (DMSO)/KOH mixture, a commercially available pseudosolvent of chitin, was capable of selectively disrupting robust cohesion between nanofibrils, as well as interlayer secondary forces between chitin molecular sheets.^[^ ^14^ ^]^ Through a sequence process of pseudosolvent swelling and maleic anhydride surface ionization, we not only realized the self‐exfoliation of chitin nanofibrils (ChNFs) in aqueous solutions, but also discovered ribbon‐like structures containing a few layered molecular sheets. This unexpected result encourages us to produce monolayered chitin SNMs by further downscaling the thickness of the above nanoribbons.

In the present work, we demonstrate that monolayered chitin nanoribbons (ChNRs) can be facilely exfoliated from their natural ingredients through a one‐pot “tandem molecular intercalation (TMI)” process under mild conditions. Relatively small‐sized DMSO first intercalates into the interlayer space of chitin molecular sheets to trigger the gap‐widening and hydroxyl activation processes, and a subsequent influx of reactive intercalate molecules (esterification reagent) with larger size thoroughly delaminate the layered structures into single molecular layered nanoribbons. The substituent groups introduced by the above modification reagents should be specially designed to meet the following criteria: 1) its size should be large enough to thoroughly eliminate the interlayer interactions between chitin molecular sheets; 2) it should contain ionogenic groups to provide sufficient repulsive force for the delamination of layered structures and stabilization of ChNRs; 3) its polarity should be close to the pseudosolvent for the acquisition of satisfactory dispersibility. After reaching the above requirements, ChNRs with an unprecedented single molecular layer of >85% (thickness <0.6 nm) and a contour length up to several micrometers are directly exfoliated in the pseudosolvent, without any extra isolation, purification, and strong postmechanical disintegration steps. In comparison with nanometric ChNFs, the size reduction of ChNRs gives them greater similarity to chitin macromolecules; the monolayered nanoribbons show crystallization‐like assembly behaviors during the drying process. Even in the solid state (e.g., mesoscale assembled films), ChNRs also represent many unique performances that rarely observed in traditional ChNF‐based materials, such as plasticization via ethanol intercalation (alcoplasticization) and self‐welding with the assistance of trace DMSO. The desirable processing and welding characteristic as well as robust mechanical strength, satisfactory water stability, and biodegradability make ChNRs’ assembly promising candidates to partially substitute petroleum‐derived and hardly degradable plastic products (e.g., highly oriented optical films and water‐stable drinking straws).

## 2 Results and Discussion

**Figure 1:**
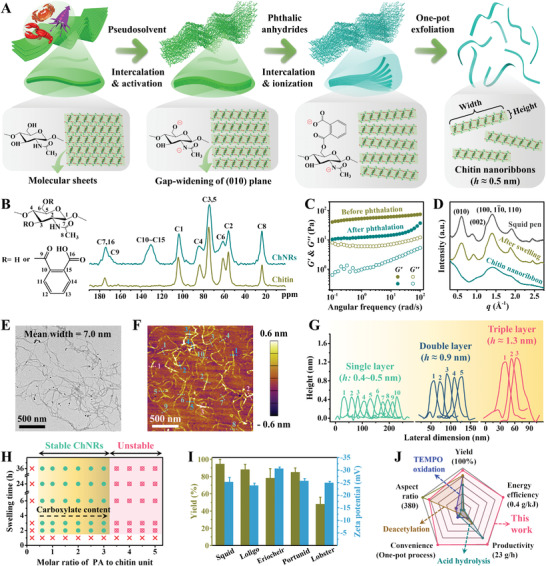
“Tandem molecular intercalation” self‐exfoliation pathway and characterization of ChNRs. A) The TMI strategy requires tandem intercalates, where DMSO first intercalates into the interlayer space of chitin molecular sheets to trigger the gap‐widening and inner hydroxyl activation processes, and then reactive anhydrides influx and thoroughly delaminate the layered structures into single molecular layered nanoribbons. B) ^13^C CP‐MAS solid state NMR, C) rheology, and D) WAXS characterization of squid *β*‐chitin before and after phthalation. E–G) Microscopic characterization of squid ChNRs: E) TEM, F) AFM, and G) corresponding height profiles along the indicated lines. H) Phase diagram for the self‐exfoliation of ChNRs as a function of swelling time in pseudosolvent and molar ratio of PA to chitin unit. I) Exfoliation yield and aqueous dispersibility of ChNRs extracted from different chitin raw materials. J) A radar chart illustrates the advantages of the TMI strategy proposed in this work.

In a typical example for the one‐pot exfoliation of monolayered ChNRs, squid pen and phthalic anhydride (PA) are selected as representative starting material and reactive intercalator, respectively (**Figure** **1**A). After a standardized purification process to remove inorganic salts and proteins, *β*‐chitin is first dispersed in the pseudosolvent of DMSO saturated with 1 mg mL^−1^ KOH under vigorous stirring. During this process, the pseudosolvent not only contributes to the high swelling and chemical activation of chitin by deprotonating its amide (—NH—CO—) and primary hydroxyl (—CH_2_—OH) groups, but also intercalates into the interlayer space of chitin molecular sheets to expand the (010) interplanar gap.^[^ ^14a^ ^]^ The former provides an efficient and homogeneous reaction environment for the surface modification, while the latter ensures the accessibility of reactive intercalator to the surface of inner (010) crystal plane. After adding PA into the above chitin suspension, a surface esterification reaction and liquid self‐exfoliation occurred simultaneously within 15 min (Figure S2, Supporting Information), leading to the highly efficient production of ribbon‐like chitin SNMs with an unprecedented >85% of single molecular layer.

The substitution reaction of the benzoic group is verified not only by the emerging chemical shifts of carbonyl ester group (C9) at 169.4 ppm and aromatic ring (C10–C15) centered at 130.9 ppm of ^3^C cross polarization/magic angle spinning (CP‐MAS) solid state nuclear magnetic resonance (NMR) in Figure 1B, but also by the Fourier transform IR (FT‐IR) signals of esterified C═O (stretching) at 1721 cm^−1^, —COO^−^ (antisymmetric stretching) at 1578 cm^−1^, aromatic ester/acid (C—O symmetric stretching) at 1258 cm^−1^, and benzene (out‐of‐plane C—H bending) at 747 cm^−1^ in Figure S3 (Supporting Information).^[^ ^15^ ^]^ The ester linkage of the large‐sized benzoic group to the surface of chitin (010) plane, on the one hand, leads to the further expansion of interplanar gap and thorough disruption of interlayer interactions. The presence of ionizable benzoic group, on the other hand, enables surface anionization of chitin molecular sheets via the deprotonation of —COOH, thus offering sufficient electrostatic repulsion for the self‐exfoliation and stabilization of ChNRs. As a result, fibrillar chitin crystals delaminate to monolayered molecular sheet and immediately disperse in the pseudosolvent of DMSO/KOH, leading to the thinning and vitrification of the suspension (Figure S4, Supporting Information). Rheological behavior of chitin/DMSO/KOH suspension before and after phthalation is further studied, and the results are illustrated in Figure 1C. The *G′* curves are fitted according to a scale equation of *G′* = *ω^n^*, where *n* can reflect the interconnected network strength formed by nanofibrils/nanoribbons.^[^ ^16^ ^]^ Compared with unreacted chitin suspensions, ChNRs show a remarkable decrease of storage modulus from 53.4 to 12.3 Pa at 1 rad s^−1^, as well as a burst increase of *n* value from 0.07 to 0.79 under the high‐frequency region, also corroborating the obvious reduction of cohesive force between nanofibrils/nanoribbons after esterification. Moreover, as shown in Figure 1D, the (010) reflection peak barely changes after pseudosolvent swelling but almost disappears after the subsequent phthalation, suggesting that most of chitin crystallites are delaminated to monolayered molecular sheet along the (010) planes. Compared with original chitin crystals, monolayered ChNRs possess greater surface disordered chains, thus showing an obvious reduction of crystallinity from 72.4% to 40.1%.^[^ ^13c^ ^]^ To be noted, we do not ascribe the decrease in crystallinity to the partially dissolution of modified chitin. This is because, on the one hand, *β*–*α* transformation of chitin barely occurs as verified by the well‐preserved merged peaks of C3 and C5 at 74.4 ppm (Figure 1B); on the other hand, the ChNRs’ suspensions (4 mg mL^−1^) exhibit gel‐like rheological behavior where *G′* is greater than *G*″ in the whole frequency range (Figure 1C). Moreover, after incubating the oven‐dried ChNRs’ films in DMSO/KOH over 3 days, the samples only moderately swell into an intact organogel rather than dissolution. Meanwhile, their dry weight shows negligible change (˂6%) compared to the original sample before swelling, further suggesting that the suspensions are basically consisted of colloidal ChNRs.

The resultant ChNRs show excellent dispersibility in the pseudosolvent of DMSO/KOH, being capable of remaining stable even for months, as well as withstanding high‐speed centrifugation (9800 rpm) or DMSO dilution process, while without giving obvious aggregation (Figure S5, Supporting Information). Such good pseudosolvent dispersibility and colloidal stability make these chitin SNMs acquirable via only one‐pot procedure and also processable for various applications. Transmission electron microscopy (TEM), scanning electron microscopy (SEM), and atomic force microscopy (AFM) characterizations shown in Figure 1E–G and in Figure S6 (Supporting Information) clearly recognize the ribbon‐like morphology of the self‐exfoliated ChNRs. They possess an average thickness of 4.8 Å with a standard deviation of 1.5 Å, an average width of 7.0 nm with a standard deviation of 1.6 nm, and an average length of 0.64 µm with a standard deviation of 0.28 µm, giving a high aspect ratio up to 671. It should be noted that a small portion of ChNRs show uneven thickness along the longitudinal direction. When carefully observing a single nanoribbon, the thickness of most areas is in the range of 3–5 Å, indicating a monolayered chitin molecular sheet, which is analogous to that of reported cellulose molecular sheet.^[^ ^13b,c^ ^]^ While, a few regions show a larger thickness of 0.8–1.3 nm, indicating the presence of 2–3 layers of molecular sheet or overlapped monolayered ChNRs. Besides, the bending and twisting of nanoribbons can also cause the locally increment of the apparent thickness. Therefore, the average thickness of a single nanoribbon is measured from AFM images based on a count of ≈10 heights at different positions along its longitudinal direction (Figure S7, Supporting Information). Meanwhile, to provide a more accurate evaluation of ChNRs’ thickness, over 60 nanoribbons are randomly determined and statistically analyzed from seven AFM images. As illustrated in Figure S8 (Supporting Information), a large number of nanoribbons are observed with a thickness of <6 Å, suggesting a high proportion (≈87.1%) of single molecular layered ChNRs. This value is not only much higher than other organic SNM suspensions,^[^ ^6^, ^9^ ^]^ but also comparable with well‐developed inorganic/carbon SNMs such as graphene and MXenes.^[^ ^3^ ^]^

The extraction conditions of ChNRs are further optimized, and its phase diagram controlled by swelling time in pseudosolvent and molar ratio of PA to chitin unit is illustrated in Figure 1H. The orthogonal experiments indicate that ChNRs cannot be well exfoliated without sufficient swelling/activation degree of chitin or substitution degree of benzoic groups. As shown in Figures S9 and S10 (Supporting Information), longer agitation time in pseudosolvent tends to disagglomerate/activate more chitin nanofibrils and thereby favors the extraction of more ChNRs, and a relatively short swelling period of 3 h is essential for the exfoliation of high‐quality monolayered chitin nanoribbons with a satisfactory yield up to 92%. We further regulate the molar ratio of PA to chitin unit from 0.1 to 5.0 while fix the swelling time (e.g., 10 h), the results demonstrate an optimized molar ratio range of 0.7–3 to achieve a maximum yield of 101.1% for single molecular layered ChNRs (Figures S11 and S12, Supporting Information). The carboxylate content of ChNRs can be facilely tuned within a range of 1.58–2.08 mmol g^−^^1^ by changing the molar ratio of PA. To be noted, in addition to the esterification of chitin molecules, PA may also be hydrolyzed to phthalic acid and thereby significantly suppress the surface ionization of exfoliated nanoribbons. As a result, ChNRs prepared under higher molar ratio (>3.5) exhibit much poorer colloidal stability, which rapidly aggregate and become weak organogel within 1 day. Thus, the molar ratio of PA to chitin unit from 0.5 to 3 is selected to exfoliate chitin nanoribbons in the following works, and the products are denoted as ChNRs‐0.5–ChNRs‐3, respectively.

Other chitin sources, such as loligo, eriocheir, portunid, and lobster, can also be utilized to produce ChNRs by experiencing the same extraction procedures, yet with different exfoliation yields (Figure 1I). These exfoliated ChNRs, though sharing a similar monolayered structure with a thickness of 4–5 Å, give different average lengths, e.g., 636 nm for squid ChNRs versus 213 nm for portunid ChNRs (Figure S13, Supporting Information). Besides being dispersed in DMSO/KOH, all these ChNRs possess good dispersity in water as well, showing a moderate zeta potential of ≈−25 mV. In Figure 1J, and Tables S1–S3 (Supporting Information), the TMI process is revealed to be capable of extracting solvent‐dispersed chitin nanomaterials in a more convenient, effective, energy‐efficient, scalable and higher‐quality manner, which is quite beneficial to the high value‐added utilization of chitin on an industrial scale.^[^ ^17^ ^]^

**Figure 2:**
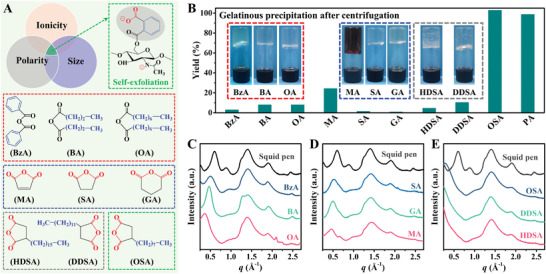
Selective criteria for reactive anhydrides applied in the TMI self‐exfoliation of ChNRs. A) Schematic diagram shows that the substituent groups introduced by reactive anhydrides have to meet the comprehensive requirements of sufficient ionicity, enough size, and matched polarity. Chemical structures of contrastive anhydrides are also illustrated and divided into four categories: BzA/BA/OA, maleic anhydride (MA)/succinic anhydride (SA)/GA (glutaric anhydride), and HDSA/DDSA just fails to introduce appropriate ionicity, size, and polarity, respectively, while PA/OSA completely reaches the above requirements. B) Exfoliation yield of ChNRs extracted from squid chitin by using different reactive anhydrides. The insets in panel (B) give the digital images of gelatinous precipitation after centrifugation, indicating the unsuccessful exfoliation of ChNRs. C–E) WAXS profiles of squid chitin before and after esterification. In all cases, the molar ratio of anhydride to chitin unit is fixed at 1.

Further investigation shows that the successful production of monolayered ChNRs depends strongly on the ionicity, size, and polarity of substituent groups (**Figure** **2**A). As illustrated in Figure 2B, both the absence of ionogenic groups (e.g., benzoic, butyric, and octanoic) and insufficient molecular size (e.g., maleic, succinic, and glutaric) result in a low exfoliation yield of ChNRs. In the case of the former anhydrides, they are capable of effectively eliminating the interlayer interactions between chitin molecular sheets, as verified by the obvious expansion of (010) interplanar spacing shown in Figure 2C. The bigger molecular size of substituent groups tends to cause a greater *d*‐spacing widening of (010) plane, e.g., 2.0 Å for benzoic anhydride (BzA), 3.2 Å for butyric anhydride (BA), and even as high as 6.8 Å for octanoic anhydride (OA), respectively. However, these groups are hard to ionize and thus cannot provide enough driving force for the spontaneous delamination of chitin crystals, as well as indispensable repulsion force for the colloidal stabilization of exfoliated ChNRs. In the case of the latter situation, fibrillar chitin crystals also fail to self‐exfoliate to monolayered molecular sheet because the interlayer interaction is still dominated by the strong cohesion. The relatively weak electrostatic repulsion provided by deprotonated carboxyl groups can only slightly enlarge the *d*‐spacing of (010) plane (Figure 2D), while, when dispersing these esterification products into NaOH aqueous solution (pH = 11), carboxyl groups fully ionize and thus significantly strengthen the electrostatic repulsion, leading to the successful self‐exfoliation of a few layered ChNRs with a high yield of 80.8–96.2% (Figure S14, Supporting Information).

To provide a deeper understanding of the self‐exfoliation mechanisms, alkyl succinic anhydrides that simultaneously meet the above requirements are further selected as the esterification reagent. As expected, the (010) reflection peak completely disappears after modification, verifying the delamination of chitin crystals to monolayered molecular sheet (Figure 2E). However, in the case of hexadecyl succinic anhydride (HDSA) and dodecyl succinic anhydride (DDSA), the exfoliation yields are as low as 4.7% and 10.6%, respectively, mainly attributed to the mismatched polarity between substituent groups and pseudosolvent. In contrast, well‐designed anhydride, such as octyl succinic anhydride (OSA) and PA, which can introduce substituent groups with appropriate ionicity, size, and polarity at the same time, is capable of realizing the self‐exfoliation of monolayered ChNRs with a high quality and yield (Figure 2B; Figure S15, Supporting Information). In addition to OSA and PA, we firmly believe that many other eligible modification reagents will be designed and applied for the extraction of single molecular layered ChNRs in the near future.

**Figure 3:**
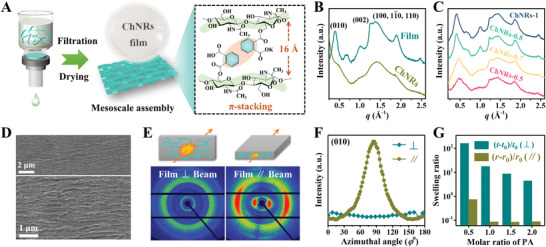
The formation of mesocrystals and recovery of crystallinity by the assembly of ChNRs. A) Procedure for improving the inter‐ChNRs’ interactions. B) WAXS profiles of lyophilized ChNRs‐1.5 floccule and assembled ChNRs‐1.5 film. C) WAXS profiles of the films assembled from ChNRs with different substitution degree of benzoic groups. D) Cross‐sectional SEM images of the assembled ChNRs‐1.5 film, which show a compact and layered structures. E) Examples of 2D WAXS scattering patterns with beam parallel and perpendicular to the film plane. F) Intensity distribution curves calculated from azimuthal integration of (010) peak from the 2D WAXS patterns in panel (E). G) Anisotropic swelling of the ChNRs’ film. *t*_0_ and *r*_0_ are the thickness and radius of the film at initial state, while, *t* and *r* are those in the swelling state at different incubation time, respectively.

The successful production of highly desirable chitin SNMs that owning unique ribbon‐like morphology, adjustable surface properties, and high‐proportioned monomolecular layer opens a new avenue for the theoretical study and practical applications of organic SNMs. For example, in comparison with nanometric ChNFs, these sub‐nanometric ChNRs exhibit crystallization‐like assembly behaviors, i.e., they are capable of coupling into single fusion mesocrystals without experiencing a melting or dissolving process. As a result, the reduction of ChNRs’ crystallinity, which is generally considered as an irreversible process, can be easily reversed. In order to promote the fusion of ChNRs, two strategies are adopted to enhance the inter‐ChNRs’ interactions (**Figure** **3**A). First, phthalic ChNRs are selected because their substitutive groups can provide strong interactions trough the *π*‐stacking of benzene rings. Next, a successive process of vacuum filtration and air‐drying is applied to densify dispersive ChNRs to dense bulk aggregate. As shown in Figure S16 (Supporting Information), the resultant ChNRs’ films are compact with a porosity of 10.1%–17.6%, together with high optical transparency (≈80% light transmittance) within the entire visible light region, indicating the uniform and close packing of ChNRs. Compared with ChNRs dried from lyophilization, the mesoscale assembled film shows a significant recovery of the crystalline structure, as clearly verified by the emerging scattering signal of (010) plane at 0.39 Å^−1^ and synchronously strengthening of other peaks intensity in Figure 3B. Due to the intercalation of large‐sized benzoic group, its (010) planar *d*‐spacing (16 Å) is much higher than that of pristine *β*‐chitin (10 Å). Moreover, higher PA concentration utilized in the modification process tends to introduce more benzoic groups on ChNRs’ surface and hereby a higher crystallinity of the assembled film (Figure 3C). The negligible variation of crystalline structure is also observed when substituting benzene rings with inactive octyl groups (Figure S17, Supporting Information). The above results clearly support that the inter‐ChNRs (010) stacking and *π*–*π* interaction are dominant in the mesoscale assembly process. The rebuilding of crystalline structure through such ChNRs’ coupling may be substantially the same phenomenon as the fusion of ultrafine cellulose nanofibrils under high temperature and pressure, lately proposed by Daicho et al.^[^ ^18^ ^]^

Further characterization by SEM, the assembled film shows a compact and isotropic morphology on the surface while straight alignment feature on the section, which is analogous to that of homogeneously dried film prepared from 2D materials (e.g., graphene and MXene) (Figure 3D; Figure S18, Supporting Information).^[^ ^19^ ^]^ Thus, we speculate that lamellate ChNRs’ mesocrystals might be first formed before stacking and fusing into a layered structure during the film‐forming process. The proposed 2D nanostructural model of ChNRs’ film is initially verified by wide‐angle X‐ray scattering (WAXS) measurements, where the incident beam parallel and perpendicular to the surface of the sample. As illustrated in Figure 3E, in the case of the beam parallel to the film, arcing scattering spots are observed in the direction perpendicular to the sample surface. It has a narrow full width at half maximum (FWHM, 38.7°) obtained from azimuthal integration of the (010) peak (Figure 3F). On the contrary, isotropic circular patterns are obtained when the film is vertically placed. Moreover, in spite of having different swelling ratio, all the ChNRs’ films demonstrate perfect anisotropic swelling behavior in DMSO, i.e., the film highly swell in the direction perpendicular to the surface, with negligible swelling along the transverse direction (Figure 3G). All these results clearly confirm the layered structures, and the thickness direction of sheet‐like assembly is perpendicular to the film surface. Higher substitution degree of benzoic groups tends to provide stronger *π*–*π* interactions and thereby a better solvent (DMSO) resistance and mechanical strength of the film (Figures S19 and S20, Supporting Information).

**Figure 4:**
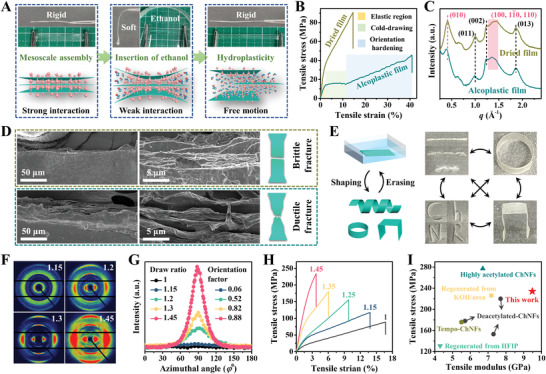
Overall plasticity of ChNRs’ films and demonstrations of their processability. A) Plasticizing process of ChNRs’ films and the corresponding schematic diagrams of interlayer expansion inducing by ethanol intercalation. B) Stress–strain curves and C) WAXS profiles of the ChNRs‐1 film before and after immerging in ethanol solutions. D) Surface and cross‐sectional SEM images of dried (top) and alcoplastic (bottom) ChNRs‐1 film after tensile failure. E) A chart and the corresponding digital images exemplifying the reversible transformation of ChNRs‐1 film from one shape to another. F) 2D WAXS profiles and G) corresponding azimuthal‐integrated intensity distribution curves of the oriented ChNRs‐1 film with draw ratios of 1.15, 1.2, 1.3, and 1.45. H) Stress–strain curves of the ChNRs‐1 film with different draw ratios. I) Ashby plot comparing the mechanical performances of chitin films prepared in this work with those acquired from literatures.

As sustainable alternatives of nonrenewable and hardly degradable petrochemicals (e.g., plastic), natural polymers (e.g., chitin, cellulose, and silk), in spite of being more available, inexpensive, environmentally friendly, and biocompatible, lack essential processability for practical applications. Although the ChNRs’ films prepared in this work show layered structures with quite weak interlayer interactions, the superimposed cohesion, over the wide contact area, is still too strong for the effortless slippage of ChNRs, namely, poor deformability and processability of the film. Here, inspired by the plasticizing process of 2D graphene, small molecular solvent is selected to weaken the inter‐ChNRs’ interactions and thereby significant facilitation of their sliding motion ability.^[^ ^20^ ^]^ To be noted, the polarity of the solvent should be meticulously controlled to ensure both the sufficient weaken of interlayer interactions and relatively low swelling ratio of the film (Figure S21A, Supporting Information). Taking comprehensive consideration of practicability and plasticizing ability, ethanol is selected to plasticize ChNRs’ films (named as alcoplastic film). The resultant sample demonstrates aspirational plasticity and thus is capable of being plastic processed to various 3D shapes, which is analogy to that applied to plastics and metals. As shown in **Figure** **4**A, after incubating in ethanol solution for ≈1 min, ChNRs’ film becomes soft, ductile, and easily processable. Its stress–strain curve presents a typical characteristic for plastic crystalline polymers, being sigmoidal in shape, as well as showing an obvious cold‐drawing plateau ranging from 2.5% to 11.4% elongation and large hysteresis loop in the loading–unloading cycles (Figure 4B; Figure S21B, Supporting Information). The ethanol molecules, on the one hand, serve as intercalator to expand the interlayer spacing between sheet‐like ChNRs’ assemblies (Figure 4C), leading to the reduction of intersheet interactions. As a result, slippage of these sheet‐like ChNRs’ assemblies, which may assume main responsibility for the cold‐drawing process, is activated. On the other hand, ethanol molecules also diffuse into the interior space of ChNRs’ assemblies. They act as a small molecular plasticizer to soften ChNRs, resulting in a decline of Young's modulus from 2.3 to 1.2 GPa, and an obvious enlargement of the orientation hardening region from 12% to 30% strain. The sustainably extension rather than immediately fracture after cold‐drawing further indicates that the sheet‐like ChNRs’ mesocrystals are firmly bridged by “amorphous” nanoribbons, in sharp contrast to those of the film assembled from individual 2D sheets (e.g., graphene and chitin nanosheets).^[^ ^20^, ^21^ ^]^ As illustrated in Figure S22 (Supporting Information), during the plastic deformation process, these ChNRs’ assemblies sequentially undergo extension of laterally linked “amorphous” nanoribbons (step I), slippage of sheet‐like mesocrystals (step II), and directional arrangement of all the nanoribbons (step III), exactly corresponding to the three distinct regions of linearly elastic, cold‐drawing, and orientation hardening in Figure 4B, respectively. Furthermore, the fracture morphology of ChNRs’ film in dried and alcoplastic state is compared in Figure 4D. In the case of the alcoplastic film, the fractured surface has a bumpy appearance with abundant micrometer‐sized cavities and pull‐out of the mesoscale layers, quite similar to the ductile fracture of cellulose nanofibrils/polymer composites.^[^ ^22^ ^]^ By contrast, cracking of the dried ChNRs’ film occurs perpendicular to the deformation direction and gives a smooth and neat cross section, indicating a relatively brittle fracture.

The reversible transition of brittle ChNRs’ film to a ductile state starts an unprecedented opportunity of plastic processing chitin into various 3D shapes. As illustrated in Figure 4E, the ChNRs’ films can be easily programmed into required shapes (e.g., helix, ring, letter, and holder) by a successive process of incubating in ethanol and air‐drying in ambient environment. These shapes are capable of remaining stable for a long time in the dry state, as well as transforming into each other arbitrarily by repeating the above process. Thanks to the relatively strong volatility of ethanol, the whole remolding process can be completed within 3 min, much faster and more convenience compared with the water‐assisted molding procedures applied to cellulose.^[^ ^23^ ^]^ Besides 3D shapes, the alcoplasticity of ChNRs’ films can also be utilized for the production of highly oriented chitin films. The rearrangement of ChNRs to an aligned state is easily realized by slowly stretching the film in ethanol solution. After drying under room temperature, a series of uniaxial oriented chitin films are prepared with a draw ratio from 1 to 1.45. The ordered structures of these films are first evaluated using 2D WAXS measurement. As illustrated in Figure 4F, in the case of relatively low draw ratio (1–1.15), isotropic scattering rings are observed, suggesting the negligible alignment of ChNRs. This result further confirms our previous point that the initial elongation of alcoplastic ChNRs’ film is mainly dominated by the slippage of sheet‐like ChNRs’ assemblies. After increasing the draw ratio from 1.15 to 1.45, the scattering signals gradually transform from rings into arcing scattering spots. The scattering spots for the (002) and (013) arcs are located on the direction of elongation, whereas those of the (010), (100), (1$\bar{1}$0), and (110) arcs are located on the perpendicular direction, clearly verifying the highly ordered arrangement of ChNRs (Figure S23, Supporting Information). The orientation factor elevates from 0.06 to 0.88 as the draw ratio increases, leading to the hardening and strengthening of ChNRs’ films (Figure 4G). As shown in Figure 4H, with an increase of draw ratio from 1.0 to 1.45, ChNRs’ films show a gradual increase of fracture strength from 88.9 to 234.1 MPa and the Young's modulus from 1.5 to 9.6 GPa. This robust mechanical performance is superior to most of reported chitin films prepared by self‐assembly of nanofibrils or regeneration of chitin solutions (Figure 4I; Table S4, Supporting Information).^[^ ^17^, ^24^ ^]^

**Figure 5:**
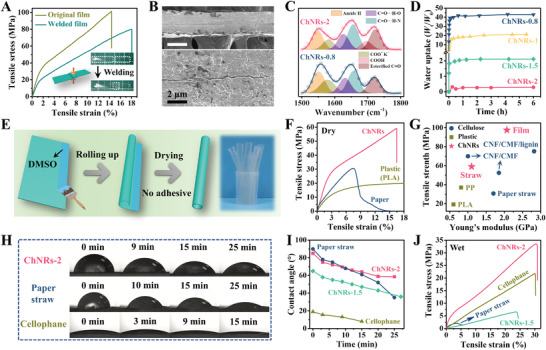
Preparation and characterization of ChNRs’ straws. A) Stress–strain curves and digital images of the intact and welded ChNRs’ films. B) Cross‐sectional SEM images of the welded region. Scale bar of the image with low magnification is 50 µm. C) FT‐IR spectra and D) water uptake–time curves of the assembled films prepared from ChNRs with different substitution degrees of benzoic groups. E) Schematic diagram of rolling up straw from ChNRs’ film. The inset shows a bunch of transparent ChNRs’ straws. F) Stress–strain curves of ChNRs, PLA, and paper straws. G) Ashby plot comparing the mechanical performances of straws obtained in this work with those acquired from literatures and commercial products. H–J) Water stability evaluation of various straws: H) digital images of contact angle test, I) contact angle–time curves, and J) stress–strain curves of the wet straws.

The fusion tendency of ChNRs not only reflects in their self‐assembly ability to form microscopic mesocrystals, but also represents on the self‐adhesive behaviors of their macroscopical film. As illustrated in **Figure** **5**A, after brushing trace DMSO on the surface of two strips end, they are overlapped and heated at 75 °C over 5 h. The DMSO treatment activates the benzoic and hydroxy groups on the film surface, forming *π*‐stacking and hydrogen‐bonding interactions that promote the interfacial adhesion between stacked ChNRs’ strips, analogy to the fusion of graphene.^[^ ^25^ ^]^ As a result, two separate ChNRs’ strips can be welded to form an integrate sample, without using any adhesives. It exhibits a tensile strength of 80.2 MPa and elongation at a break of 18.1%, which are close to those of the pristine ChNRs’ film. Further investigation of welded region by SEM shows negligible gap between the two separate samples, clearly confirming the satisfactory self‐adhesiveness of the ChNRs’ films (Figure 5B). In addition to provide strong adhesive force via *π*–*π* interactions, the hydrophobic phenyl groups also endow ChNRs’ films with excellent water stability. Although highly hydrophilic carboxylates are simultaneously introduced after phthalation, they are capable of in situ converting to uncharged carboxylic groups by the neutralization reaction with phthalic acid (by‐product) (Figure 5C; Figure S24, Supporting Information). Therefore, higher substitution degree of benzoic groups results in a lower water uptake of the ChNRs’ films. Impressively, the ChNRs‐2 film shows only 29% mass increase after incubating in water for 6 h, nearly seven times less than that of unmodified *β*‐chitin film prepared from KOH/urea solution (Figure 5D).^[^ ^26^ ^]^

The combination of plasticity, self‐adhesive ability, and water stability offers the facileness of processing ChNRs’ film to various practical products. For example, a straw can be easily produced by rolling up a rectangular ChNRs’ film on a glass rod and then welding its edges via trace DMSO, as illustrated in Figure 5E. It shows high stiffness with a Yong's modulus of 1.1 GPa, satisfactory robustness with a tensile strength of 59.1 MPa, and desirable deformability with a fracture strain of 16.5%, which are not only superior to the mechanical performances of commercial straws (e.g., polypropylene, polylactic acid, and paper), but also comparable with state‐of‐the‐art biobased straws prepared from cellulose nanofibrils (Figure 5F,G).^[^ ^27^ ^]^ We further immerge these straws in water over 4 h to evaluate their water stability. As shown in Figure S25 (Supporting Information), the ChNRs’ straw shows negligible absorbed water beyond the liquid level, and substantially remains integrity of their structure and even welding region, suggesting an excellent wetting and water stability. Its surface contact angle retains a high value of 58.5° after a 25 min test, which are 1.7 and 7.3 times higher than that of commercial paper straws and cellophane, respectively (Figure 5H,I). Therefore, even after incubating in water for ≈30 min, the wet ChNRs’ straw still demonstrates good mechanical performance with a tensile strength up to 33.5 MPa and elongation at a break of 30.4%, outperforming those of the commercial paper straws (Figure 5J).

## 3 Conclusion

In conclusion, monolayered ChNRs, a new kind of organic SNMs, are successfully produced by using the top‐down liquid exfoliation approach through a TMI process. TMI requires tandem intercalates, where DMSO first intercalates into the interlayer space of chitin molecular sheets to trigger the gap‐widening and inner hydroxyl activation processes, and then reactive anhydrides influx and thoroughly delaminate the layered structures into single molecular layered nanoribbons. Any anhydrides that can introduce substituent groups with sufficient ionicity, enough size, and matched polarity are capable of realizing the one‐pot extraction of ribbon‐like chitin SNMs with a high yield of nearly 100% and a high portion (>85%) of single molecular layer (thickness <0.6 nm). In comparison with their nano‐counterparts (ChNFs), these ChNRs exhibit a series of unique properties owing to their sub‐nanometric size and rich surface functional groups. In the case of colloidal state, ChNRs are capable of coupling into single fusion mesocrystals and thus recovering their lowered crystallinity during the film‐forming process. The mesoscale‐assembled films also represent intriguing performances that rarely observed in traditional ChNF‐based films, such as plasticization via ethanol intercalation and self‐welding with the assistance of trace DMSO. The desirable processing and welding characteristics as well as robust mechanical strength, satisfactory water stability, and biodegradability make ChNRs’ assembly promising candidates to partially substitute petroleum‐derived and hardly degradable plastic products (e.g., highly oriented optical films and water‐stable drinking straws). The finding in this work not only offers a low‐cost, energy‐efficient, convenient, effective, and scalable platform for the production of high‐quality monolayered ChNRs, but also opens a new avenue for both the theoretical study and practical applications of organic SNMs.

## Conflict of Interest

The authors declare no conflict of interest.

## Supporting information

Supporting Information

## Data Availability

The data that support the findings of this study are available from the corresponding author upon reasonable request.
