# Supplementary material for: Single Molecular Layer of Chitin Sub‐Nanometric Nanoribbons: One‐Pot Self‐Exfoliation and Crystalline Assembly into Robust, Sustainable, and Moldable Structural Materials
Source: Adv Sci (Weinh). 2022 Mar 31;9(16):2201287. doi: 10.1002/advs.202201287 (PMC9165516; doi:10.1002/advs.202201287)
Supplement: Supplementary file 1 — Supporting Information [file ADVS-9-2201287-s001.pdf]

Supporting Information

Single Molecular Layer of Chitin Sub-Nanometric Nanoribbons: One-Pot Self-Exfoliation and Crystalline Assembly into Robust, Sustainable and Moldable Structural Materials.

By *Yugao Ding*[1], *Xizhi Chen*[1], *Youshuang Zhou*[1], *Xiaoming Ren*[1], *Weihua Zhang*[3], *Mingjie Li*[3], *Qunchao Zhang*[1], *Tao Jiang*[1], *Beibei Ding*[2]*, *Dean Shi*[1]* *and Jun You*[1]*

[*] Prof. J. You, Prof. D. Shi, Prof. T. Jiang, Prof. Q. Zhang, Prof. X. Ren, Y. Zhou, X. Chen, Y. Ding

[1]Key Laboratory for the Green Preparation and Application of Functional Materials, Hubei Key laboratory of Polymer Materials, School of Materials Science and Engineering, Hubei University, Youyi Road 368, Wuhan 430062, China.

E-mail: yjgreen123@hubu.edu.cn; deanshi2012@yahoo.com

Dr. B. Ding

[2]Key Laboratory for Deep Processing of Major Grain and Oil, Wuhan Polytechnic University, Ministry of Education, Wuhan 430023, China

E-mail: dingbb@whpu.edu.cn

W. Zhang, Prof. M. Li

[3]CAS Key Lab of Bio-based Materials, Qingdao Institute of Bioenergy and Bioprocess Technology, Chinese Academy of Sciences, Songling Road 189, Qingdao 266101, P. R. China

Keywords: chitin nanoribbons; pseudo-solvent; self-exfoliation; single molecular layer; sub-nanometric materials

Experimental section

Supporting figures

Table S1, S2, S3, S4

Figure S1-Figure S25

References S1-S13

Experimental Section

Material

Eriocheir shells and squid pen were purchased from Gold-Shell Pharmaceutical Co. Ltd. (Zhejiang, China). Loligo pen, portunid and lobster shells were collected from local seafood market. A standardized purification procedure was applied to these raw materials to remove minerals and proteins according to our previous work[S1]. The DD of original α-chitin (eriocheir shell) and β-chitin (squid pen) is determined by potentiometric titration to be 3.4% and 3.3%, respectively. DMSO, acid anhydrides, KOH and other reagents were purchased from Sinopharm Chemical Reagent Co. Ltd. (Beijing, China) and used without purification. Deionized water was utilized in the whole work.

Self-exfoliation of chitin nanoribbons

Pseudosolvent (DMSO saturated with 1 mg/mL KOH) was used to swell purified chitin under room temperature. The concentration of chitin was fixed at 4 mg/mL for β-chitin and 20 mg/mL for α-chitin, respectively. After agitating for designed duration (1~36 h for β-chitin and 24 h for α-chitin), phthalic anhydride (PA/chitin structural unit = 0.1~5, mol/mol) was added and intensely stirred for another 15 min. The resultant viscous suspensions were diluted four times with pure DMSO (1 mg/mL for β-chitin and 5 mg/mL for α-chitin), followed by centrifugation at 9500 rpm for 20 min to remove the un-exfoliated samples. The supernatant transparent suspensions, which contained a high portion of monolayered ChNRs were collected and stored at ambient environment (~20 ^{o}C). In order to make the subsequent structural characterization (FT-IR, WAXS and solid-state 13C NMR) more accurate, ChNRs were precipitated and repeatedly centrifugally washed (CENCE H1850, Hunan, China) with ethanol. Then, dried and purified samples were obtained by solvent exchange to tertiary butanol and subsequent freeze-drying. The absence of FT-IR signals at 1850

and 1780 cm^{-1} in Figure S2B, Figure S3, Figure S9B and Figure S11B confirm that the purified ChNRs are free of the unreacted phthalic anhydride. The clean NMR spectrum shown in Figure S11D further supports the above viewpoint.

Fabrication of ChNRs films, straws and their mechanics performance testing

ChNRs films were prepared by a successive process of filtering the suspension through 220 nm pore filter membranes and drying at 70 ^{o}C over 6 h. Oriented ChNRs films were obtained by stretching the native film in ethanol solution to a designed draw ratio (1~1.45). Rectangular strips were cut from dried ChNRs film and rolled up on a glass stick. The edges of the samples were brushed by trace DMSO and then sealed by pressing to adhere. After heating at 75 ^{o}C over 5 h, the joints were welded and the intact straws could be easily separated from the glass stick. Tensile stress-strain curves were recorded on an Electromechanical Universal Testing Machine (INSTRON 68TM-10) at an elongation rate of 2 mm/min. The Young's modulus and toughness were calculated from the initial slope and the lower area from tensile stress-strain curves.

Characterization

Transmission electron microscopy (TEM) images of ChNRs were recorded on a Hitachi TEM (H-7650) instrument operating at an accelerating voltage of 80 kV. Atomic force microscope (AFM) measurements were used to accurately evaluate the thickness of monolayered ChNRs, using an Agilent 5400 in an intermittent mode at a scan rate of 1 Hz. TEM and AFM samples were prepared by dripping a drop of diluted ChNRs suspensions (dispersed in the pseudosolvent of DMSO/KOH) on the surface of a copper grid and a clean mica sheet, respectively, followed by vacuum-drying under 60 ^{o}C. Scanning electron microscopy (SEM) measurements were performed on a Field Emission Scanning Electron Microscope (Hitachi S4800, Japan) operating at a voltage of 3 kV. The samples were treated with sputtering platinum before observation. Before SEM test, the

ChNRs suspensions were firstly concentrated into an organogel by vacuum-filtration using a microporous membrane with a pore size of ~220 nm. The SEM samples were then obtained by solvent exchange to tertiary butanol and subsequent freeze-drying. Moreover, the successful and efficient interception of ChNRs further indicate that the modified chitin is in colloidal state rather than molecular state (dissolved in the pseudosolvent).

2D WAXS profiles of all the samples were recorded on a Small Angle X-ray scatterometer (Xeuss 2.0) using a 2D-sensor and Genix 3D X beamline with wavelength (λ) of 1.54 Å. The sample-to-detector distance was fixed at 151.7 mm. The corresponding 1D WAXS curves were obtained by circularly averaging the intensity by the build-in software. In order to prevent the self-assembly of ChNRs, the samples were directly precipitated by ethanol, followed by tertiary butanol-exchange and freeze drying. In the case of ethanol treated ChNRs film (alcoplastic film), the sample was totally exchanged to t-BuOH before undergoing a freezing-dry process with liquid nitrogen and a freezer dryer. FT-IR measurements were performed on a Nicolet iS50 Fourier transform infrared spectrometer. Zeta potential of ChNRs aqueous suspensions (0.1 mg/mL, pH = 10.5) was recorded on a Zatasizer Nano-ZS90 (Malven Instruments, UK) at 25 °C. The carboxylate contents of the ChNRs were tested by the electrical conductivity titration method. Before the zeta-potential and titration measurements, the solvent of DMSO was exchanged to aqueous solution via prolonged dialysis. Light transmission of ChNRs suspensions and films was evaluated on a Shimadzu UV3600 UV-Vis spectrophotometer using a quartz cuvette with an optical path of 1 cm.

Rheology measurements were performed on a controlled stress/strain rheometer (TA discovery HR2) with a cone-plate geometry. Dynamic frequency sweep test (0.1 to 100 rad/s) was carried out to compare the rheology behavior of β-chitin/DMSO/KOH suspension (4 mg/mL) before and after phthalation. For each measurement, the strain amplitude was set as 10% and the temperature was fixed at 25 ^{o}C. Water contact angle was tested with a water droplet of fixed 2 μL on a drop shape

analysis system (JC2000D1). The reported values were calculated by averaging values measured at five different surface locations. Solid-state ^{13}C NMR spectra of ChNRs were recorded on a JNM-ECZ600R/S3 spectrometer operated at a ^{13}C frequency of 100 MHz. The experimental parameters were set as the following: the spinning speed of 12 kHz, the contact time of 5 ms, the acquisition time of 50 ms and the recycle delay of 2 s. The carboxylate content of ChNRs were determined by titration. The Herman's orientation factor (*f*) was calculated from the azimuthal profile according to the equation of $f = \frac{3\langle cos^2\gamma\rangle - 1}{2}$. The average cosine $\langle cos^2\gamma\rangle$ was obtained from the following equation: $\langle cos^2\gamma\rangle = 1 - 2\langle cos^2\emptyset\rangle$, where $\langle cos^2\emptyset\rangle = \frac{\int I(\emptyset)\cos^2\emptyset \sin\emptyset \, d\emptyset}{\int I(\phi)\sin\emptyset \, d\emptyset}$.

Table S1. Calculation of energy-consumption and time-consumption for different chitin exfoliation procedures during the pre-treatment process.

Treatment of 1 kg raw chitin		Concentration (wt%)	Pre-treatment														
				Pseudo-solvent treatment					Ionization								
				Mechanical agitation					Mechanical agitation					Heating		
			Total volume (L)	Power (W)	Period (h)	Capacity (L)	Time consumption (h)	Energy consumption (kJ)	Power (W)	Period (h)	Capacity (L)	Time consumption (h)	Energy consumption (kJ)	Power (W)	Time consumption (h)	Energy consumption (kJ)
TEMPO oxidation	α-chitin	1	100.0	/	/	/	/	/	16	1.5	20	7.5	432.0	/	/	/
	β-chitin	1	100.0	/	/	/	/	/	16	2	20	10.0	576.0	/	/	/
Deacetylation	α-chitin	4	25.0	/	/	/	/	/	16	2	20	2.5	144.0	600	2.5	5400
	β-chitin-1	2.5	40.0	/	/	/	/	/	16	12	20	24.0	1382.4	/	/	/
	β-chitin-2	/	/	/	/	/	/	/	/	/	/	/	/	/	/	/
Acid hydrolysis	α-chitin	3.3	30.3	/	/	/	/	/	16	6	20	9.1	523.6	600	9.1	19656
	β-chitin	3.3	30.3	/	/	/	/	/	16	1.5	20	2.3	130.9	600	2.3	4968
Tandem molecular intercalation	α-chitin	2	50	16	12	20	30	1728	16	0.25	20	0.6	36.0	/	/	/
	β-chitin	0.4	250	16	3	20	37.5	2160	16	0.25	20	3.1	178.6	/	/	/

Table S2. Calculation of energy-consumption and time-consumption for different chitin exfoliation procedures during the disintegration process.

Treatment of 1 kg raw chitin		Concentration (wt%)	Disintegration process													
				Homogenization				Probe sonication				Mechanical agitation				
			Total volume (L)	Power (W)	Effeciency (mL/min)	Time consumption (h)	Energy consumption (kJ)	Power (W)	Effeciency (mL/min)	Time consumption (h)	Energy consumption (kJ)	Power (W)	Period (h)	Capacity (L)	Time consumption (h)	Energy consumption (kJ)
TEMPO oxidation	α-chitin	0.1	1000.0	/	/	/	/	300	100	166.7	180000.0	/	/	/	/	/
	β-chitin	0.2	500.0	200	200	41.7	30000	300	100	83.3	90000.0	/	/	/	/	/
Deacetylation	α-chitin	0.3	333.3	/	/	/	/	400	40	138.9	200000.0	/	/	/	/	/
	β-chitin-1	0.3	333.3	1500	166.7	33.3	179946	/	/	/	/	/	/	/	/	/
	β-chitin-2	0.2	500.0	/	/	/	/	300	50	166.7	180000.0	/	/	/	/	/
Acid hydrolysis	α-chitin	1.5	66.7	/	/	/	/	800	100	11.1	32000.0	/	/	/	/	/
	β-chitin	1.5	66.7	/	/	/	/	300	40	27.8	30000.0	/	/	/	/	/
Tandem molecular intercalation	α-chitin	1	100.0	/	/	/	/	/	/	/	/	/	/	/	/	/
	β-chitin	0.2	500.0	/	/	/	/	/	/	/	/	/	/	/	/	/

Table S3. Calculation of total energy-consumption and time-consumption for different chitin exfoliation procedures.

Treatment of 1 kg raw chitin		Total energy consumption (kJ)	Total time consumption (h)	Yield (%)	Mass of obtaied nanofibrils (kg)	Energy consumption per mass (kJ/g)	Conversion rate (g/h)	Ref.
TEMPO oxidation	α-chitin	180432	174.2	90	0.9	200.5	5.17	S2
	β-chitin	120576	135	70	0.7	172.3	5.19	S3
Deacetylation	α-chitin	205544	141.4	90	0.9	228.4	6.36	S4
	β-chitin-1	181328.4	57.3	64.5	0.645	281.1	11.26	S5
	β-chitin-2	180000	166.7	100	1	180.0	6.00	S6
Acid hydrolysis	α-chitin	52179.6	25.8	60	0.6	87.0	23.26	S7
	β-chitin	35098.9	44	60	0.6	58.5	13.64	S8
Tandem molecular intercalation	α-chitin	1764.0	30.625	85	0.85	2.1	27.76	This work
	β-chitin	2338.6	40.625	92	0.92	2.5	22.65	This work

Table S4. Mechanical properties of different chitin films.

Source	Method	Ultimate tensile Strength (MPa)	Strain-to-failure (%)	Modulus (GPa)	Toughness (MJ/m^3)	Transmittance @ 600 nm (%)	Ref.
Chitin	Deacetylation	153	8	7.3	8.3	68	S9
	Deacetylation	220	7.3	7.7	10.4	90	S5
	Deacetylation	178	8.3	5.7	9.9	/	S10
	Highly acetylated ChNFs	277	7.1	6.7	13.2	94	S10
	Regenerated from KOH/urea	226	13	7.2	20.3	92	S11
	Regenerated from HFIP	131	14.2	4.3	/	92	S12
	TEMPO-ChNFs	176	5.4	5.5	/	/	S13
	Tandem molecular intercalation	234.1	3.7	9.6	52.3	98	This work

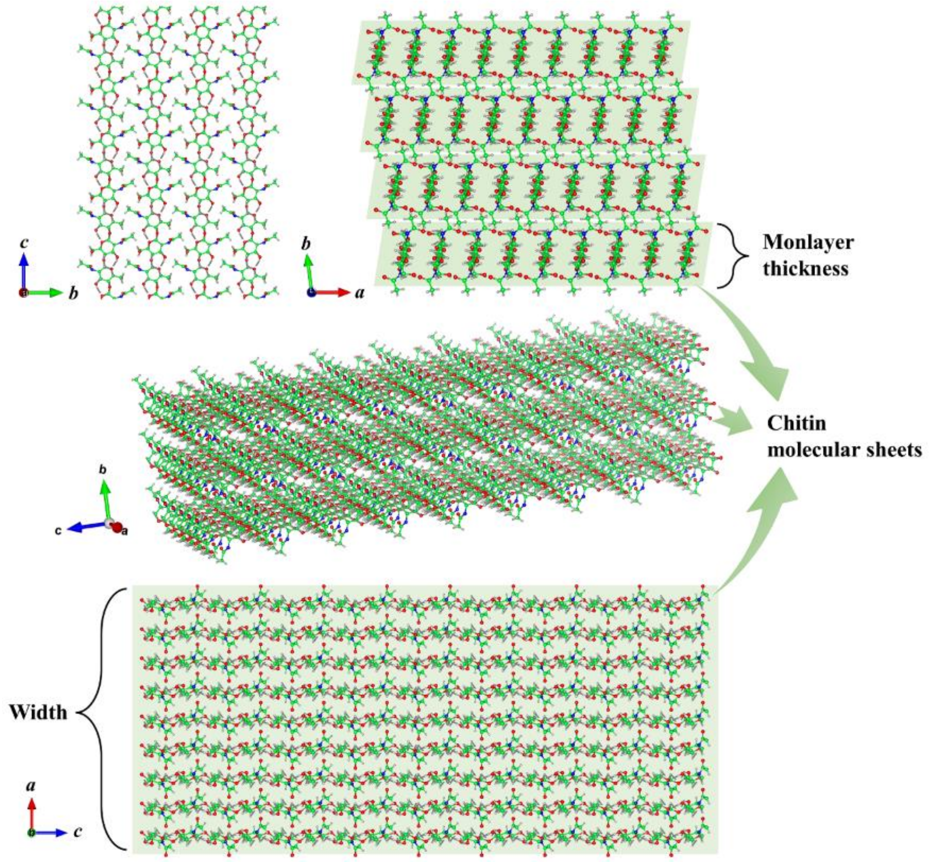

Figure S1. Molecular models of the crystal structure of β-chitin showing the lamellar structures consists of monolayered molecular sheets.

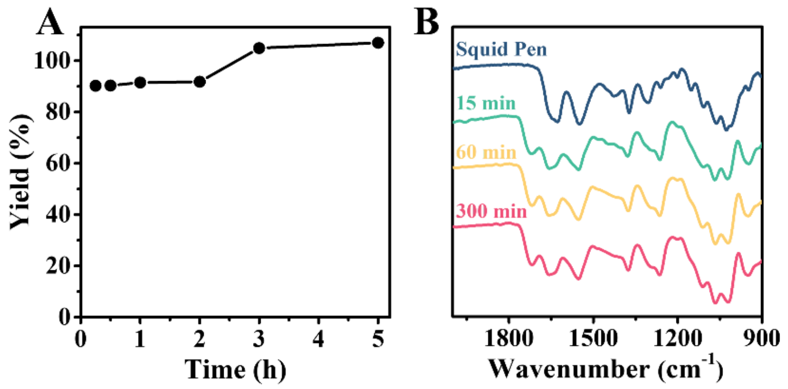

Figure S2. A) Yield of ChNRs as a function of esterification times. B) FT-IR spectra of squid ChNRs prepared with different esterification times.

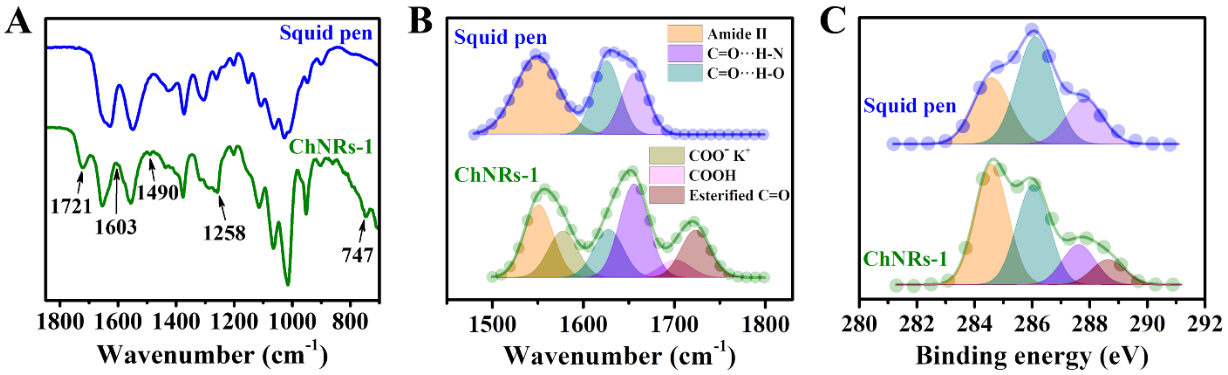

Figure S3. FT-IR and XPS analysis of squid chitin and ChNRs.

The weak absorbances at 1603 and 1490 cm^{-1} seen in Figure S3A are attributed to the stretching vibration of aromatic ring skeleton (C=C). Figure S3C illustrates the curve fitting of high-resolution multiplex scan spectra of the C1s region of squid pen and ChNRs. Three peaks at 284.6, 286 and 287.5 eV are observed in the spectrum of squid pen, corresponding to the carbon of alkyl (C-C/C-H), alcoholic/etheric/amine (C-O-C/C-OH/C-N) and acetamide (C=O) groups, respectively. After esterification, the peak at 284.6 eV dramatically increases due to the introduction of abundant aromatic nucleus (C=C), which also have strong signal at 284.6 eV. Moreover, a new peak at 288.8 eV, which attributed to the carbon of carboxyl groups (-COOH), emerges in the spectrum of ChNRs. These results further verify the successful linkage of benzoic group to chitin chains.

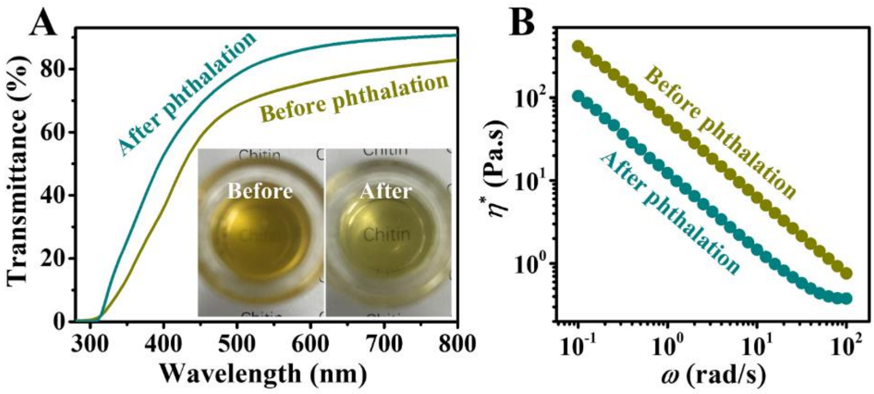

Figure S4. UV-vis transmittance spectra (A) and complex viscosity (B) of squid chitin/DMSO/KOH suspension before and after phthalation.

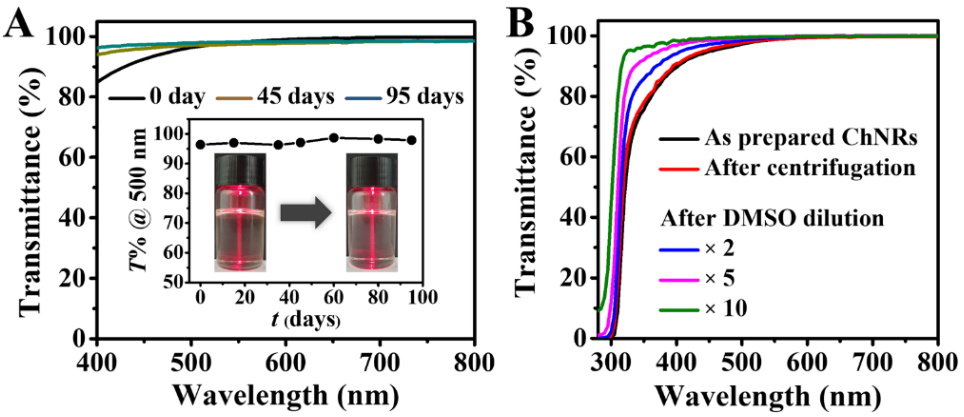

Figure S5. A) Referenced transmittance history of squid ChNRs suspension from 0 to 95 days, illustrating the excellent stability of ChNRs in the pseudosolvent. B) UV-vis transmittance spectra of squid ChNRs suspensions before and after high-speed centrifugation (9800 rpm) or DMSO dilution.

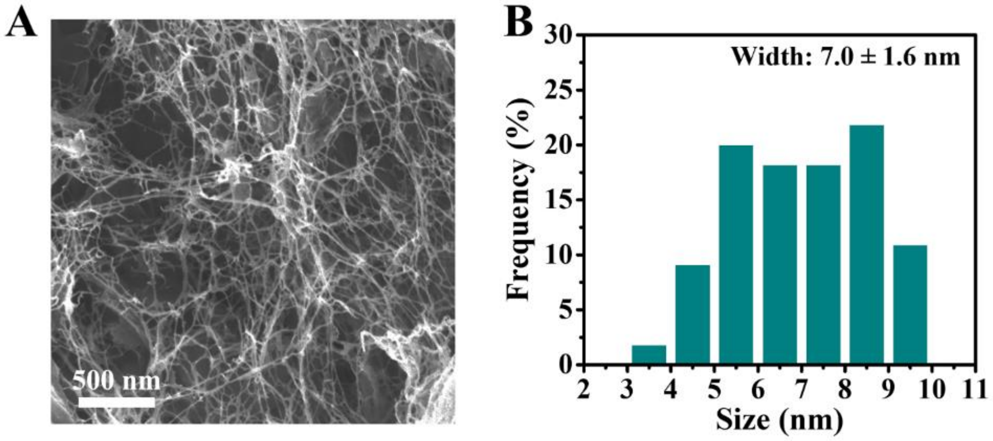

Figure S6. A) SEM image of ChNRs extracted from squid pen. B) ChNRs wide histograms obtained from Figure 1E.

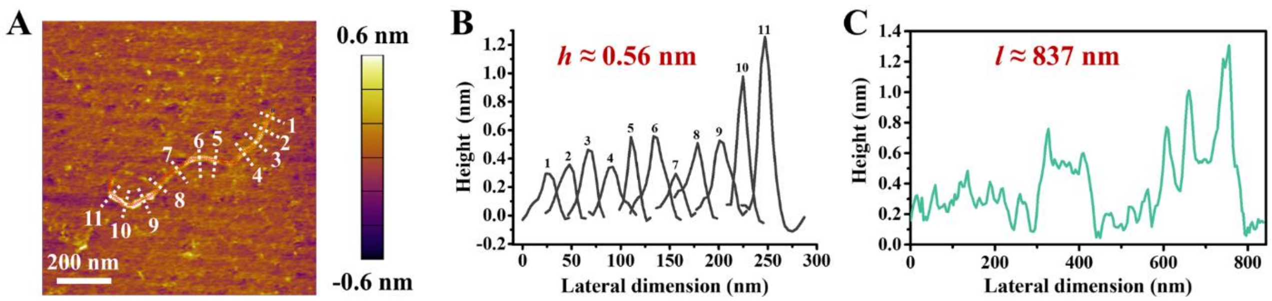

Figure S7. A typical example shows how to obtain the average thickness of a single nanoribbon. It is measured from AFM images based on a count of ~10 heights at different positions along its longitudinal direction.

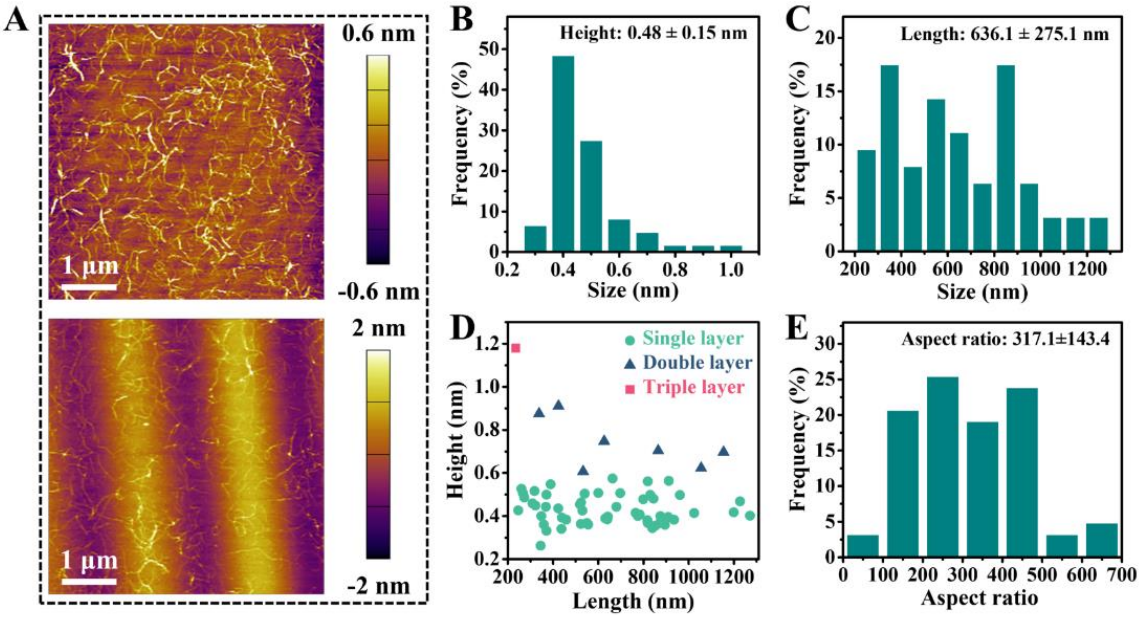

Figure S8. A) AFM images of ChNRs-2 extracted from squid pen. B-E) ChNRs-2 size distribution analyzed from seven AFM images.

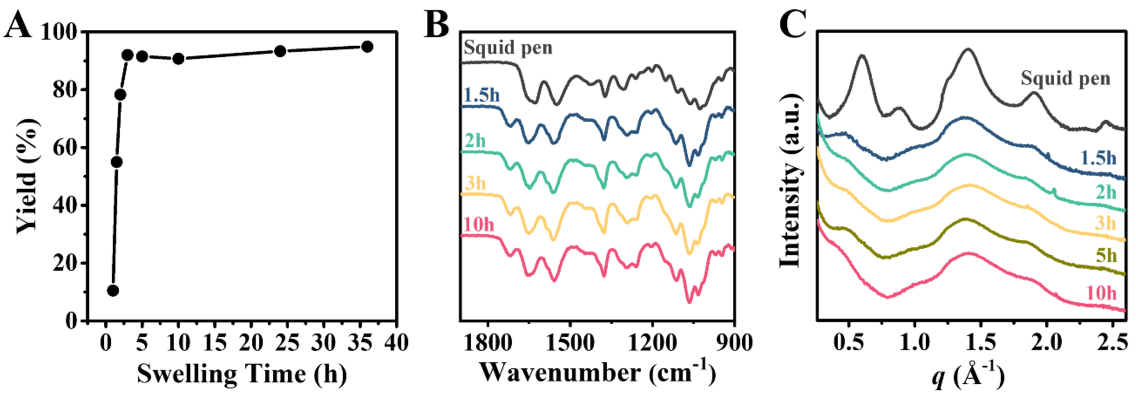

Figure S9. A) Exfoliation yield of squid ChNRs as a function of swelling time. B-C) FT-IR spectra and WAXS profiles of squid ChNRs prepared with different swelling times (molar ratio of PA to chitin unit is fixed at 1).

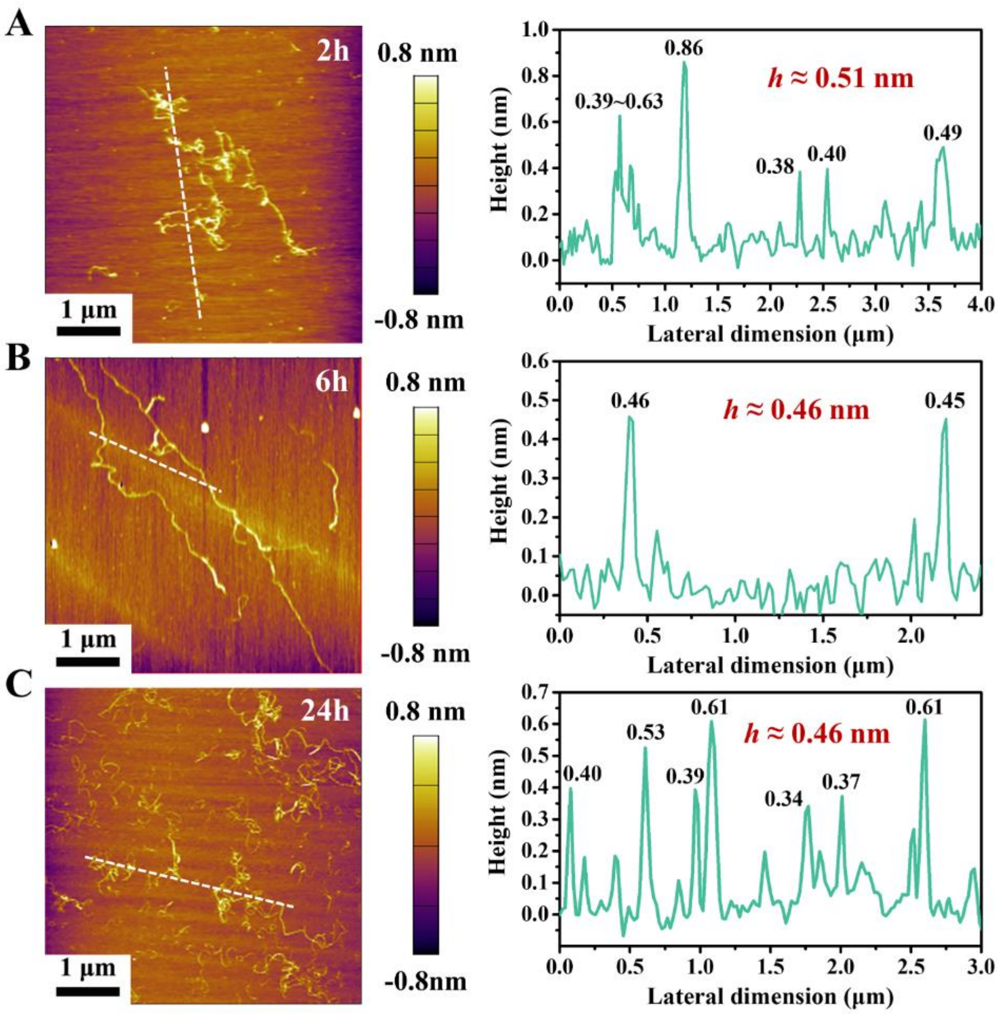

Figure S10. AFM images and corresponding height histograms of squid ChNRs prepared with different swelling durations.

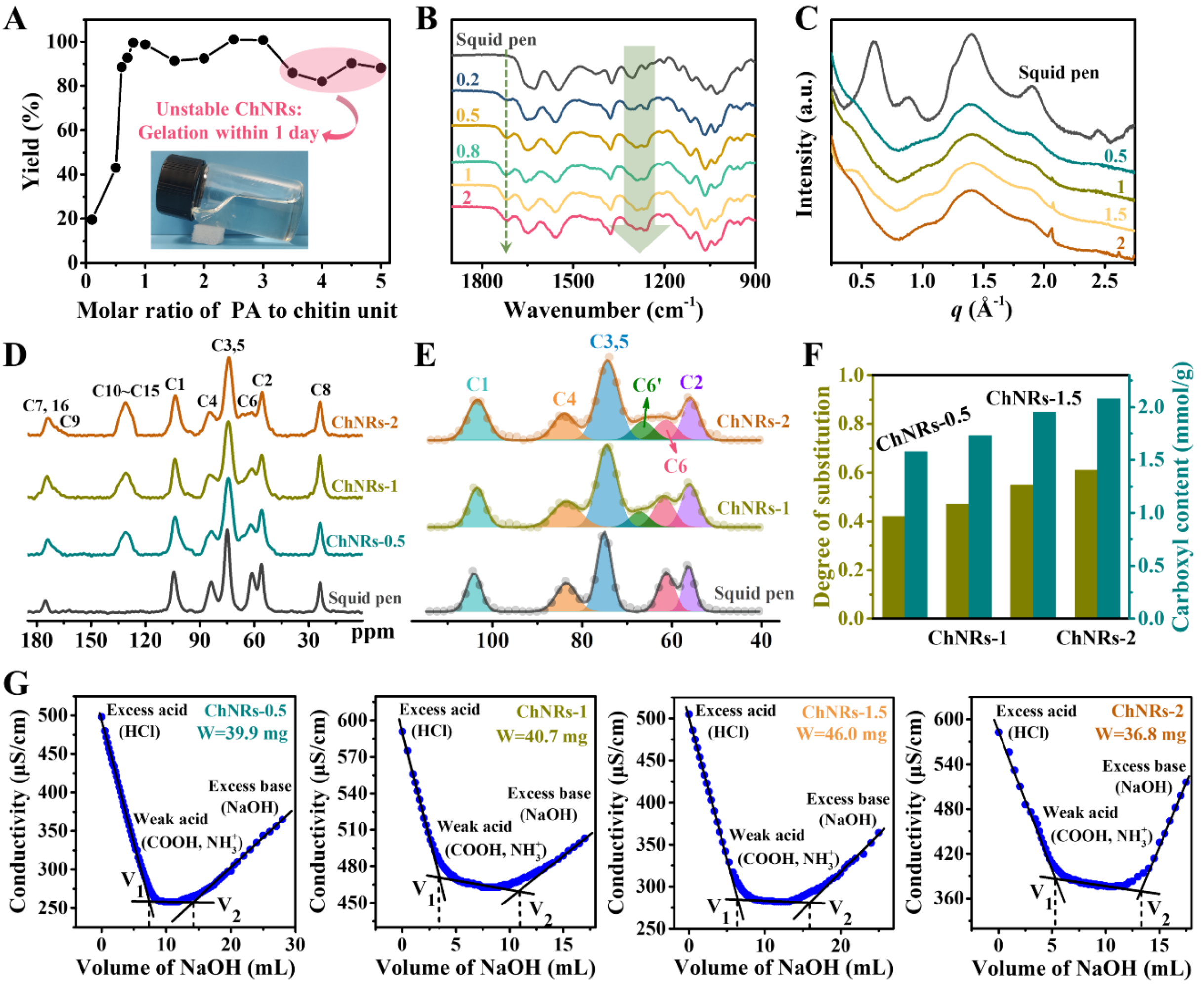

Figure S11. A) Exfoliation yield of squid ChNRs as a function of PA molar ratio. B-G) FT-IR spectra, WAXS profiles, ^{13}C CP-MAS solid state NMR spectra, titrimetric curves, degree of substitution and carboxyl content of squid ChNRs prepared with different molar ratio of PA (swelling time of chitin in the pseudosolvent is fixed at 12h).

Titrimetric method is selected to determine the carboxylate content of PA-esterified chitin samples, which are further used for the calculation of accurate DS values. The solvent of DMSO was firstly exchanged to aqueous solution via prolonged dialysis. Then a small amount of 1 M HCl was added to the suspension to set the conductivity in the range of 500~600 µs/cm. A 0.01 M NaOH solution was slowly added and the conductivity variation of the ChNRs suspension was monitored by using a conductance titrator. The obtained conductivity-NaOH dosage curve reflect the content of both carboxylate (CC) and C2 amino groups (AC) in the ChNRs (Figure S11G).

Thus, CC+AC can be calculated according to the equation of CC+AC=c_{NaOH}×(V_2-V_1)/W. Moreover, the degree of deacetylation (DD) of squid chitin is determined to be 3.3% and it barely change during the esterification process, as verified by the FT-IR and NMR results. Therefore, DS values of ChNRs can be further obtained according to the following equation: (DS+DD)×1000/(203+148.1×DS-42×DD)=CC+AC. Finally, the DS values are calculated to be 0.42, 0.47, 0.55 and 0.61, namely, a carboxylate content of 1.58, 1.73, 1.95 and 2.08 mmol/g for ChNRs-0.5, ChNRs-1, ChNRs-1.5 and ChNRs-2, respectively.

Moreover, as illustrated in Figure S11E, with the aiding of peak-differentiating and imitating, C6 (C6 carbons bearing unsubstituted) and C6' (C6 carbons bearing substituted) can be clearly identified. The peak intensity of C6 (61.2 ppm) is found to become weaker, whereas a new peak at 66.3 ppm (C6') appears and gradually strengthens with an increase of total DS value. DS value of C6 can be estimated from the relative integral intensities of peaks C6 and C6'. Then, DS value of C3 can be further calculated from the total DS value obtained by the titrimetric analysis and the DS of C6. By above method, DS values of C6/C3 are determined to be 0.34/0.08, 0.35/0.12 and 0.49/0.12 for ChNRs-0.5, ChNRs-1 and ChNRs-2, respectively. The order of reactivity is C6>C3, which is similar to that observed from modification of chitin/cellulose under both homogeneous and heterogeneous environments.

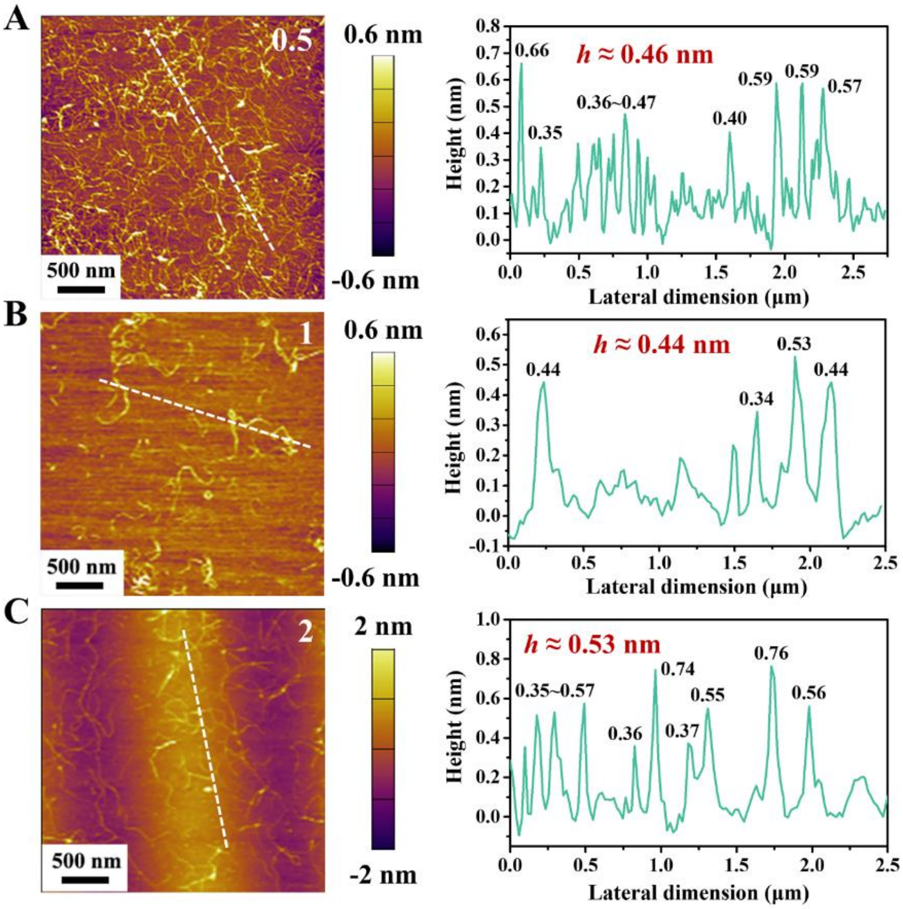

Figure S12. AFM images and corresponding height histograms of squid ChNRs prepared with different molar ratio of PA to chitin unit.

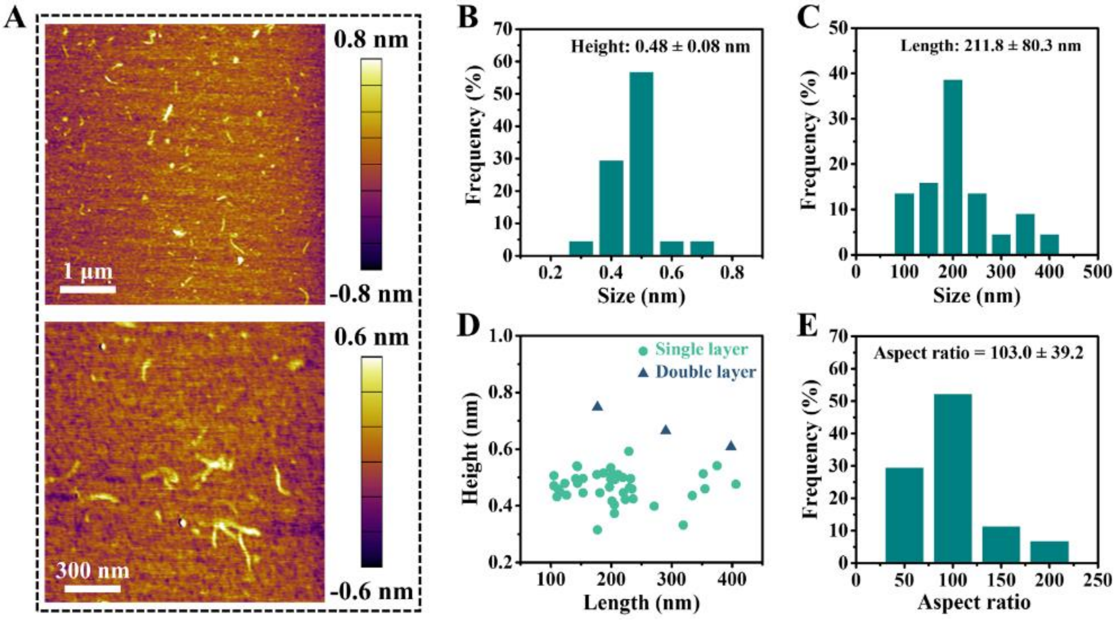

Figure S13. A) AFM images of ChNRs-2 extracted from portunid shells. B-E) ChNRs-2 size distribution analyzed from six AFM images.

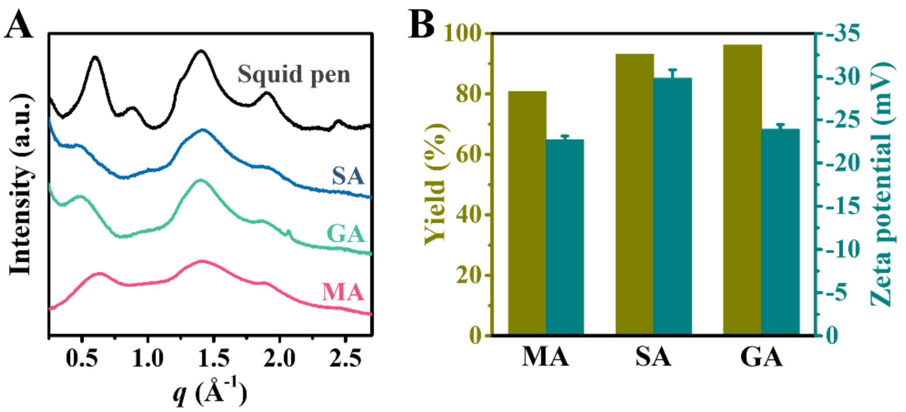

Figure S14. WAXS profiles, aqueous solutions exfoliation yield and zeta potential of ChNRs prepared by using SA, GA and MA as reactive intercalator, respectively.

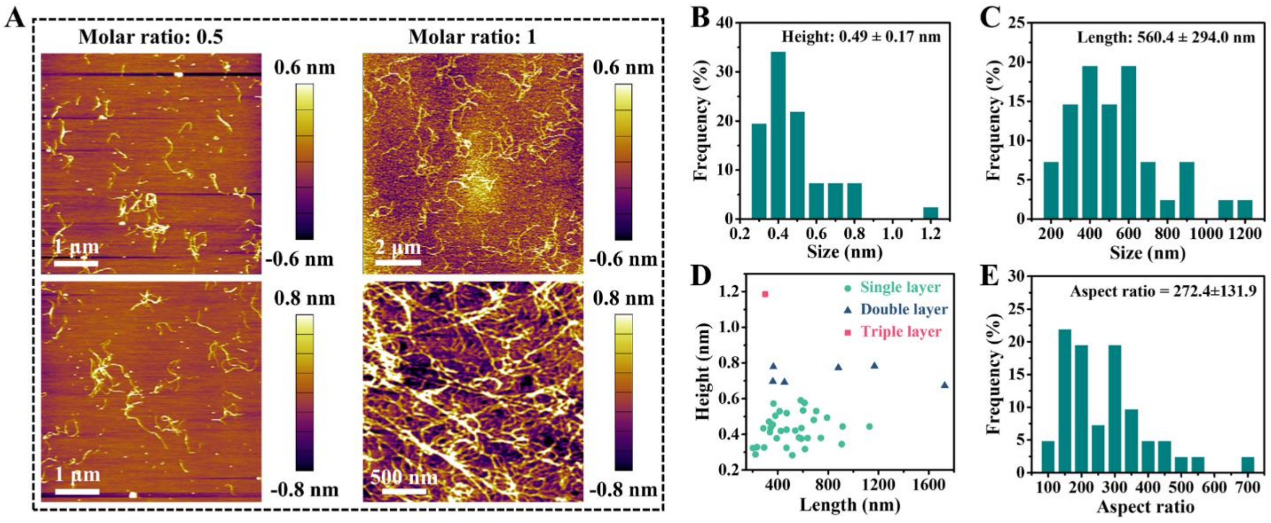

Figure S15. A) AFM images of ChNRs-0.5 and ChNRs-1 prepared by using OSA as reactive intercalator. B-E) ChNRs-0.5 size distribution analyzed from six AFM images.

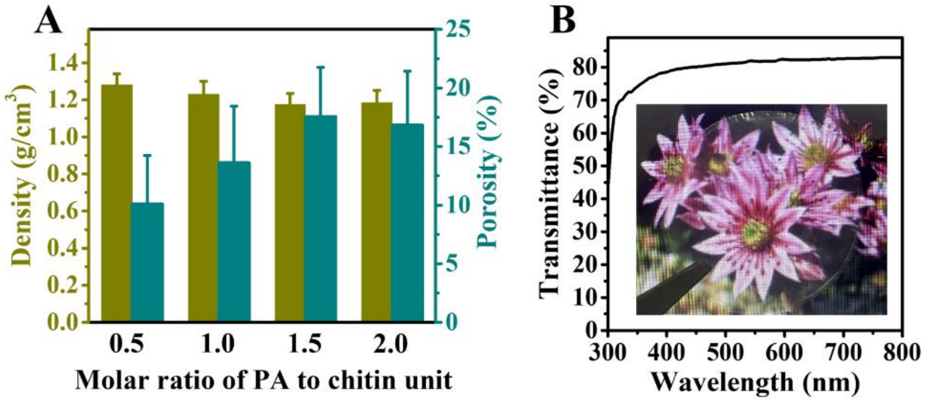

Figure S16. A) Density and porosity of the ChNRs-1 films at relative humidity of 41% and temperature of 25 ^oC. B) UV-vis transmittance of ChNRs film. The inset shows the film transparency.

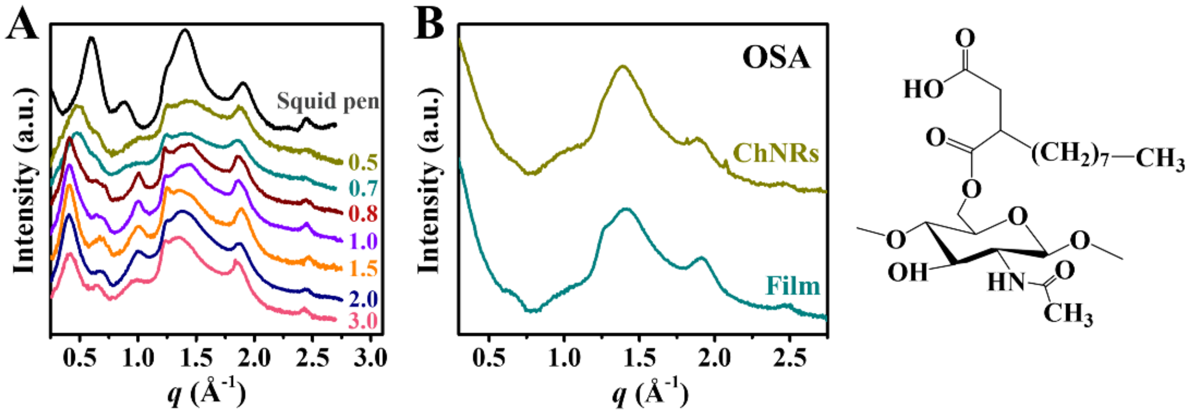

Figure S17. A) WAXS profiles of the films assembled from ChNRs with different substitution degree of benzoic groups. B) WAXS profiles of the films assembled from ChNRs prepared by using OSA as reactive intercalator.

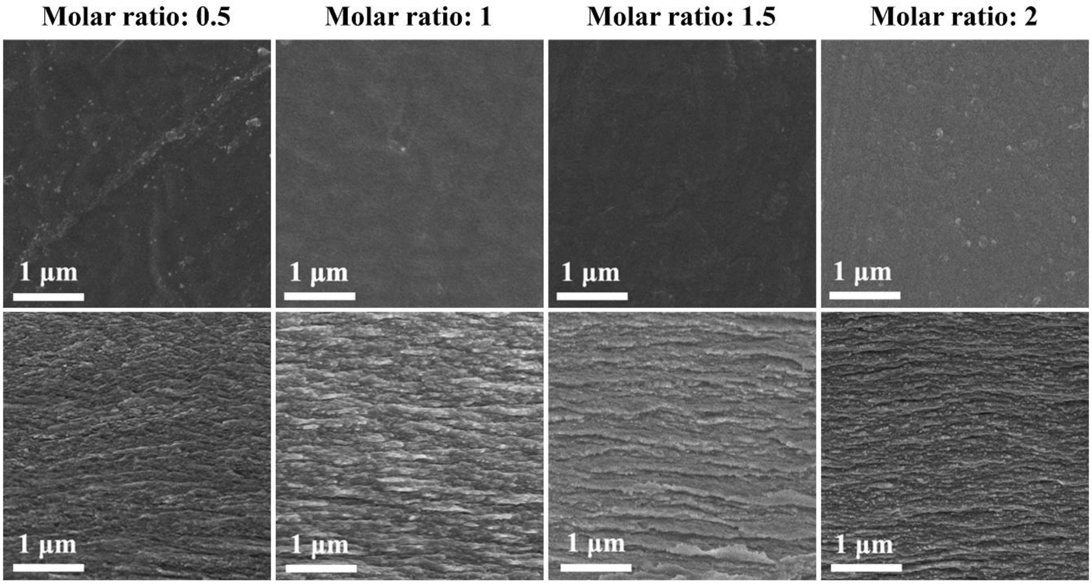

Figure S18. Surface (up) and cross-sectional (bottom) SEM images of ChNRs films.

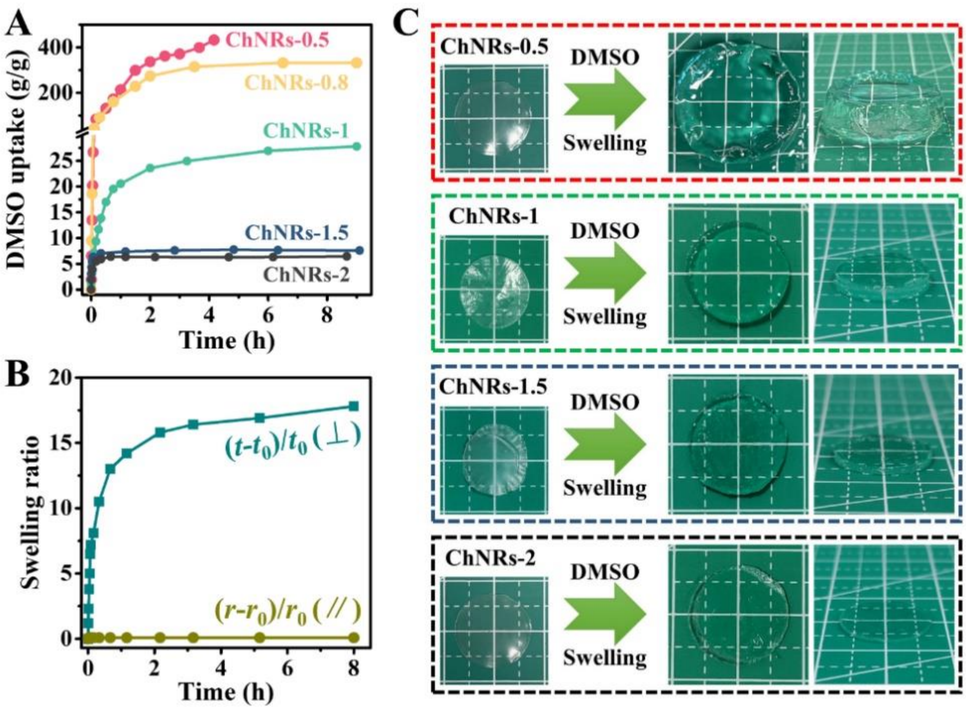

Figure S19. A) DMSO uptake-time curves for the films assembled from ChNRs with different substitution degree of benzoic groups. B) Swelling ratio-time curve of ChNRs-1 film. C) Digital images of ChNRs films before and after swelling in DMSO for approximately 8h.

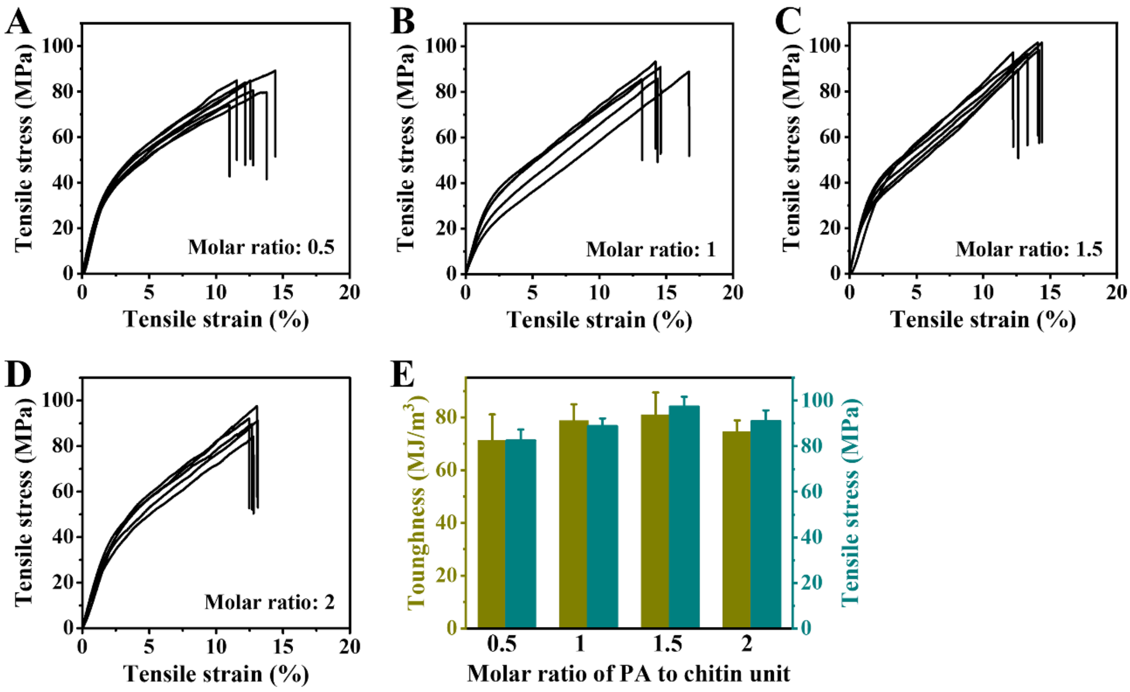

Figure S20. A-D) Stress-strain curves of the films assembled from ChNRs with different substitution degree of benzoic groups: 0.5 (A), 1 (B), 1.5 (C) and 2 (D). E) Average toughness and tensile stress of these ChNRs films.

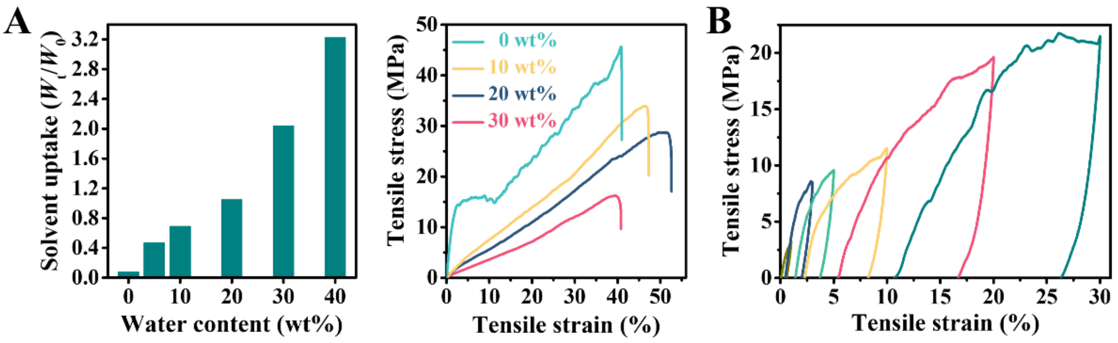

Figure S21. A) Solvent uptake and stress-strain curves of the ChNRs-1 film after immerging in ethanol/water mixed solutions with different water content. B) Loading-unloading cycles of the alcoplastic ChNRs film with corresponding stretch ratio.

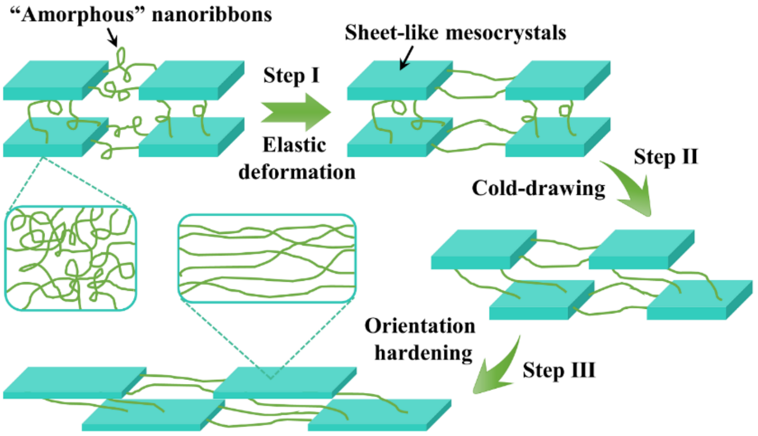

Figure S22. Structural evolution of alcoplastic ChNRs film during the deformation process.

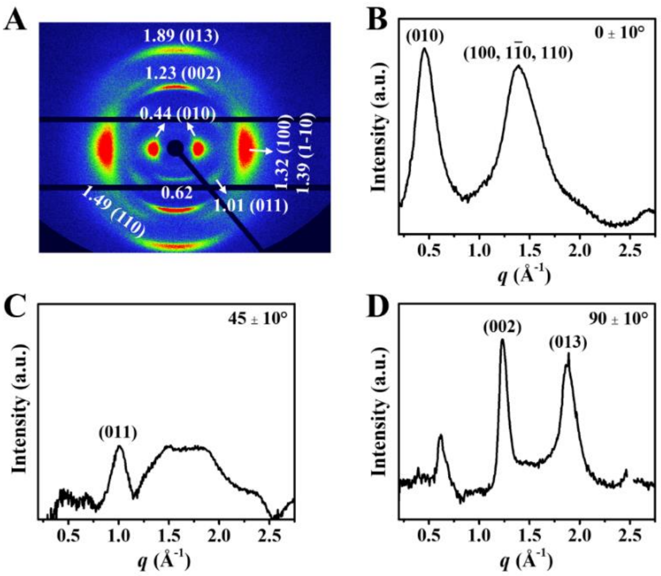

Figure S23. A) 2D WAXS patterns of oriented ChNRs film. B-D) WAXS profiles according to the azimuthal area of interest: (B) represent data obtained for φ = 0 ± 10º, (C) represent data obtained for φ = 45 ± 10º, (D) represent data obtained for φ = 90 ± 10º.

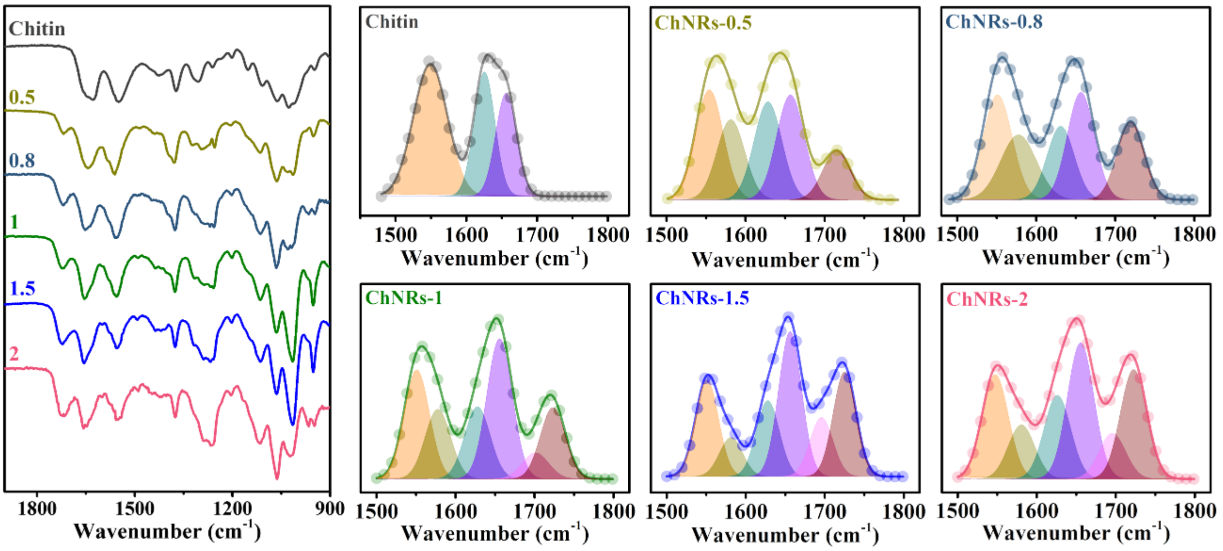

Figure S24. FT-IR spectra of the assembled films prepared from ChNRs with different substitution degree of benzoic groups.

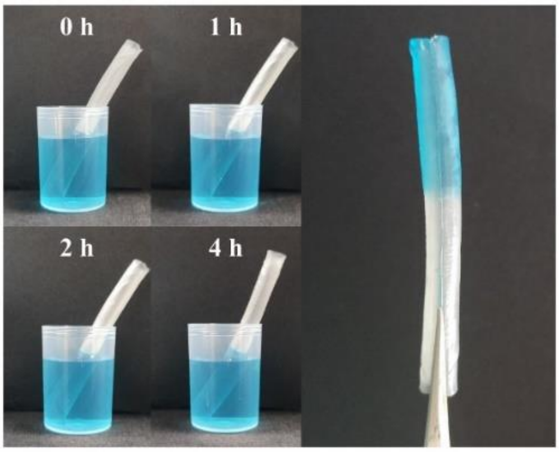

Figure S25. Water immersing test for ChNRs-2 straw.

Reference

[S1] J. You, L. Zhu, Z. Wang, L. Zong, M. Li, X. Wu, C. Li, *Chem. Eng. J.* **2018**, *344,* 498.

[S2] Y. Fan, T. Saito, A. Isogai, *Biomacromolecules* **2008**, *9*, 192.

[S3] Y. Fan, T. Saito, A. Isogai, *Carbohydr. Polym.* **2009**, *77*, 832.

[S4] Y. Fan, T. Saito, A. Isogai, *Carbohydr. Polym.* **2010**, *79*, 1046.

[S5] Q. Wu, N. E. Mushi, L. A. Berglund, *Biomacromolecules* **2020**, *21*, 604.

[S6] Y. Fan, T. Saito, A. Isogai, *Biomacromolecules* **2008**, *9*, 1919.

[S7] Y. Huang, M. Yao, X. Zheng, X. Liang, X. Su, Y. Zhang, A. Lu, L. Zhang, *Biomacromolecules* **2015**, *16*, 3499.

[S8] M. Paillet, A. Dufresne, *Macromolecules* **2001**, *34*, 6527.

[S9] N. E. Mushi, N. Butchosa, M. Salajkova, Q. Zhou, L. A. Berglund, *Carbohydr. Polym.* **2014**, *112*, 255.

[S10] Q. Wu, E. Jungstedt, M. Šoltésová, N. E. Mushi, L. A. Berglund, *Nanoscale* **2019**, *11*, 11001.

[S11] J. Huang, Y. Zhong, L. Zhang, J. Cai, *Adv. Funct. Mater.* **2017**, *27*, 1701100.

[S12] J. Jin, D. Lee, H. Im, Y. Han, E. Jeong, M. Rolandi, K. Choi , B. Bae. *Adv. Mater.* **2016** , *28*, 5169.

[S13] Q. Wu, J. Engström, L. Li, H. Sehaqui, N. Mushi, L.Berglund. *ACS Sustainable Chem. Eng.* **2021**, *9*, 5356.
